# TSIE: Robust Blind Locomotion Learning for Bipedal Robots via Terrain and State Implicit-Explicit Estimation

**DOI:** 10.3390/biomimetics11090615

**Published:** 2026-09-01

**Authors:** Zhiyuan Liang, Jie Xue, Haiming Mou, Qingdu Li, Jianwei Zhang

**Affiliations:** 1 School of Optical-Electrical and Computer Engineering, University of Shanghai for Science and Technology, Shanghai 200093, China; 231260251@st.usst.edu.cn (Z.L.); 221240078@st.usst.edu.cn (J.X.); 2 Institute of Machine Intelligence, University of Shanghai for Science and Technology, Shanghai 200093, China; 3 Shanghai DroidUp Co., Ltd., Shanghai 200433, China; mhming@droidup.com; 4 Department of Informatics, University of Hamburg, 20146 Hamburg, Germany; jianwei.zhang@uni-hamburg.de

**Keywords:** bipedal robot, robust blind locomotion, state implicit-explicit estimation, terrain implicit-explicit estimation, multi-scale terrain representations

## Abstract

Robust blind locomotion over complex unstructured terrains relies on accurate estimation of robot states and surrounding terrain geometry. However, under real-world deployment conditions without exteroceptive perception, it remains challenging to accurately estimate robot states and infer surrounding terrain structures solely from noisy proprioceptive observations. Existing methods commonly learn single-scale implicit terrain representations from historical proprioceptive observations and explicitly estimate robot states. However, they lack explicit terrain estimation and may lose critical geometric details. Moreover, single-scale terrain information is insufficient to capture both local geometric structures and global terrain trends. To address these issues, we propose a Terrain and State Implicit-Explicit Estimation (TSIE) framework to improve the locomotion capability of bipedal robots over complex terrains. TSIE encodes long-horizon proprioceptive observations using a Long Short-Term Memory (LSTM) network and introduces a dual-branch architecture consisting of a Terrain Implicit-Explicit Estimator (Terrain-IE) and a State Implicit-Explicit Estimator (State-IE). Terrain-IE performs multi-scale terrain implicit-explicit estimation by explicitly estimating a local high-resolution height map and implicitly reconstructing a global low-resolution height map. By preserving gradient connections between the two terrain branches, Terrain-IE enables joint implicit-explicit training of multi-scale terrain representations, improving terrain understanding and estimation accuracy. State-IE explicitly estimates the base linear velocity and foot-centered height map, while implicitly reconstructing future proprioceptive states to further improve tracking performance and locomotion robustness. We validate TSIE through simulation and real-world experiments on a full-sized bipedal robot platform with a height of 170cm and a mass of 35kg. Experimental results show that TSIE outperforms baseline methods in complex-terrain traversal capability, terrain and state estimation accuracy, and velocity-tracking stability. Real-world deployment further demonstrates the robustness of TSIE across indoor and outdoor complex terrains, achieving a 95% success rate in continuous stair ascent and descent tasks.

## 1. Introduction

Bipedal robots have human-like morphologies and can traverse unstructured terrains such as stairs, slopes, and steps, showing great potential in industrial inspection and disaster rescue [1]. To address model uncertainties and external disturbances in robotic systems, robust iterative learning control has been integrated with neural network compensation to improve the robustness of uncertain nonlinear robotic systems [2]. Meanwhile, reinforcement learning (RL) has achieved significant progress in legged locomotion control by leveraging massively parallel simulation, domain randomization, and end-to-end policy optimization [3,4,5,6]. However, compared with wheeled and quadrupedal robots, bipedal robots have a smaller support area, a higher center of mass, more frequent contact transitions, and more strongly coupled dynamics, making robust locomotion over complex terrains particularly challenging [7,8,9,10].

Under the reinforcement learning framework, bipedal locomotion control is commonly formulated as a Partially Observable Markov Decision Process (POMDP). During real-world deployment, robots can only access proprioceptive observations provided by joint encoders and inertial measurement units, while privileged information such as base linear velocity, contact forces, terrain height maps, friction coefficients, and external disturbances is difficult to obtain directly [11]. Although exteroceptive sensors such as depth cameras and LiDARs can provide terrain information, they are susceptible to sensor noise, occlusions, latency, calibration errors, and illumination variations, which may reduce the reliability of high-frequency closed-loop control [12,13].

From the perspective of biomimetic engineering design, biological adaptive capabilities can be abstracted at the functional and behavioral levels and translated into engineering implementations in robotic systems [14]. Inspired by the human ability to adapt to terrain through proprioception and body–environment interactions under limited visual perception, proprioception-based blind locomotion provides a deployment-efficient and robust control paradigm for traversing complex terrain.

Compared with exteroception-based methods, estimating low-dimensional states such as base linear velocity, base height, and foot contact states from proprioceptive observations is relatively tractable. However, terrain structures can only be inferred indirectly from foot contacts and robot state responses [15,16], making direct estimation of high-dimensional terrain height maps highly challenging. To alleviate this issue, existing methods usually encode terrain information or all privileged information into low-dimensional implicit features from historical proprioceptive observations, while explicitly estimating key states such as base velocity to improve locomotion stability over complex terrains [11,17,18,19,20]. Nevertheless, compressing privileged information into low-dimensional implicit features may lose critical geometric details of the terrain and makes it difficult to distinguish the different roles of terrain, velocity, contact, and disturbance information in locomotion control [18,21,22].

Furthermore, existing implicit terrain reconstruction methods typically rely on single-scale terrain height map representations [18,23], which are insufficient to capture both local geometric details and global terrain trends. Specifically, local high-resolution height maps can characterize fine structures around the feet, such as stair edges, obstacle heights, and feasible foothold regions, but they lack the capability to model overall terrain variations. In contrast, global low-resolution height maps can reflect large-scale terrain undulations and trends, but they tend to lose critical local geometric details. For terrains with abrupt height changes, such as stairs, discrete obstacles, and uneven steps, the policy needs to perceive both local structures and global trends to adjust gait rhythm and foothold strategies in advance [21]. Therefore, single-scale terrain representations are insufficient for robust blind locomotion over complex unstructured terrains.

Based on the above analysis, improving robust blind locomotion of bipedal robots over complex terrains requires joint modeling of multi-scale terrain geometry and key motion states. To this end, we propose a reinforcement learning framework based on Terrain-State Implicit-Explicit Estimation, enabling the policy to obtain more informative terrain and state representations without exteroceptive perception. The proposed framework relies solely on historical proprioceptive observations and uses a LSTM network to extract implicit dynamic features from robot–environment interactions. It further introduces a dual-branch implicit-explicit estimation architecture consisting of Terrain-IE and State-IE. Terrain-IE integrates local high-resolution explicit terrain estimation with global low-resolution implicit terrain reconstruction to capture both local geometric details and global terrain trends. State-IE combines explicit velocity estimation, foot-centered height map estimation, and future proprioceptive state reconstruction to enhance the policy’s modeling capability for motion states, contact transitions, and external disturbances.

The main contributions of this work are summarized as follows:

1. We propose TSIE, an end-to-end framework for robust blind bipedal locomotion, which jointly estimates multi-scale terrain and state information solely from proprioceptive histories.
2. We design Terrain-IE, which integrates local explicit height map estimation with global implicit height map reconstruction, and introduce adaptive sampling (AdaSmpl) to improve training stability.
3. We design State-IE, which combines explicit velocity estimation, foot-centered height map estimation, and future proprioceptive state reconstruction to enhance the policy’s state estimation capability.
4. We validate the effectiveness of TSIE through simulation and real-world robot experiments. The results show that TSIE outperforms baseline methods in terrain traversal capability, estimation accuracy, velocity tracking, and generalization over complex terrains.

## 2. Related Work

### 2.1. Legged Robot Locomotion Learning

Compared with conventional approaches based on dynamic modeling and adaptive control [24,25], deep reinforcement learning leverages large-scale simulation, domain randomization, and end-to-end optimization, demonstrating stronger adaptability in complex-terrain locomotion control for legged robots [5,6]. To improve training efficiency and real-world robustness, existing methods commonly exploit privileged information during simulation training, such as terrain height, ground friction, and external disturbances. These methods mainly fall into two categories: two-stage teacher–student frameworks [26] and single-stage asymmetric Actor-Critic frameworks [5].

Two-stage teacher-student frameworks first train a teacher policy with access to full simulation states or environmental information, and then transfer the teacher’s action distribution or latent representations to a student policy that relies only on proprioception through imitation learning [20,26]. Such methods can exploit privileged information to improve environmental adaptability, but the discrepancy between teacher and student inputs may lead to representation mismatch and covariate shift, thereby degrading robustness during real-world deployment [18]. In contrast, single-stage asymmetric Actor-Critic frameworks utilize privileged information within a unified reinforcement learning process. In this paradigm, the Actor only uses deployable proprioceptive observations, while the Critic accesses full privileged information during training to guide policy optimization. This avoids representation transfer issues introduced by two-stage distillation and has become an important training paradigm in legged robot reinforcement learning [17,27,28].

### 2.2. Terrain and State Estimation Methods

How to leverage proprioceptive histories within a single-stage reinforcement learning framework and transform privileged information available during training into deployable terrain and state representations is a key problem in blind locomotion over complex terrains. Recent studies commonly introduce state estimation, privileged information encoding, or world-model reconstruction modules into asymmetric Actor-Critic frameworks, enabling the policy to infer robot states, terrain properties, and external disturbances from limited proprioceptive observations, thereby improving velocity tracking performance and locomotion robustness.

Ji et al. [27] jointly trained the policy network and a state estimator to explicitly estimate key states such as base linear velocity. Nahrendra et al. [17] employed a context-aided estimation network to estimate linear velocity and reconstruct future states, enabling the policy to implicitly infer terrain properties from proprioceptive histories. Gu et al. [19] used a denoising world model learning (DWL) to reconstruct privileged information, including terrain and robot states, from noisy observations. Ma et al. [18] proposed the VPIES framework, which compressed terrain and dynamics information into latent features through a privileged encoder, guiding the policy to infer terrain features and estimate linear velocity from dual-timescale proprioception. In addition, Wang et al. [29] quantitatively evaluated different state estimation variables and showed that linear velocity and foot-height maps are key estimates for complex-terrain locomotion, further validating the effectiveness of their combination in real-world deployment.

Although these methods improve proprioception-based blind locomotion, they still have several limitations. Existing proprioception-based terrain estimation and privileged-information encoding methods typically learn single-scale implicit terrain representations to facilitate policy optimization. However, they lack explicit modeling of terrain geometry and are unable to simultaneously capture local geometric details and global terrain trends. To address these limitations, we propose the TSIE framework, which employs an implicit-explicit estimation mechanism to jointly recover multi-scale implicit-explicit terrain representations, latent robot dynamics, and key motion states from historical proprioceptive observations. This enables the unified modeling of local explicit terrain, global implicit terrain, latent dynamics, and key robot states within a single-stage reinforcement learning framework, providing more accurate environmental and state representations for robust blind locomotion over complex terrains.

## 3. Method

The proposed TSIE framework is a single-stage end-to-end reinforcement learning framework, in which the neural network directly generates desired joint target positions from the robot’s proprioceptive observations. TSIE avoids the representation mismatch commonly introduced by teacher–student frameworks and mitigates the loss of geometric information caused by purely implicit terrain feature extraction in single-stage learning frameworks. By leveraging long-horizon proprioceptive memory features and a dual-branch implicit-explicit estimation module composed of Terrain-IE and State-IE, TSIE aims to improve the estimation of key robot states and surrounding terrain geometry. We first provide an overview of the framework, followed by a detailed description of each component.

### 3.1. Framework Overview

**Figure 1:**
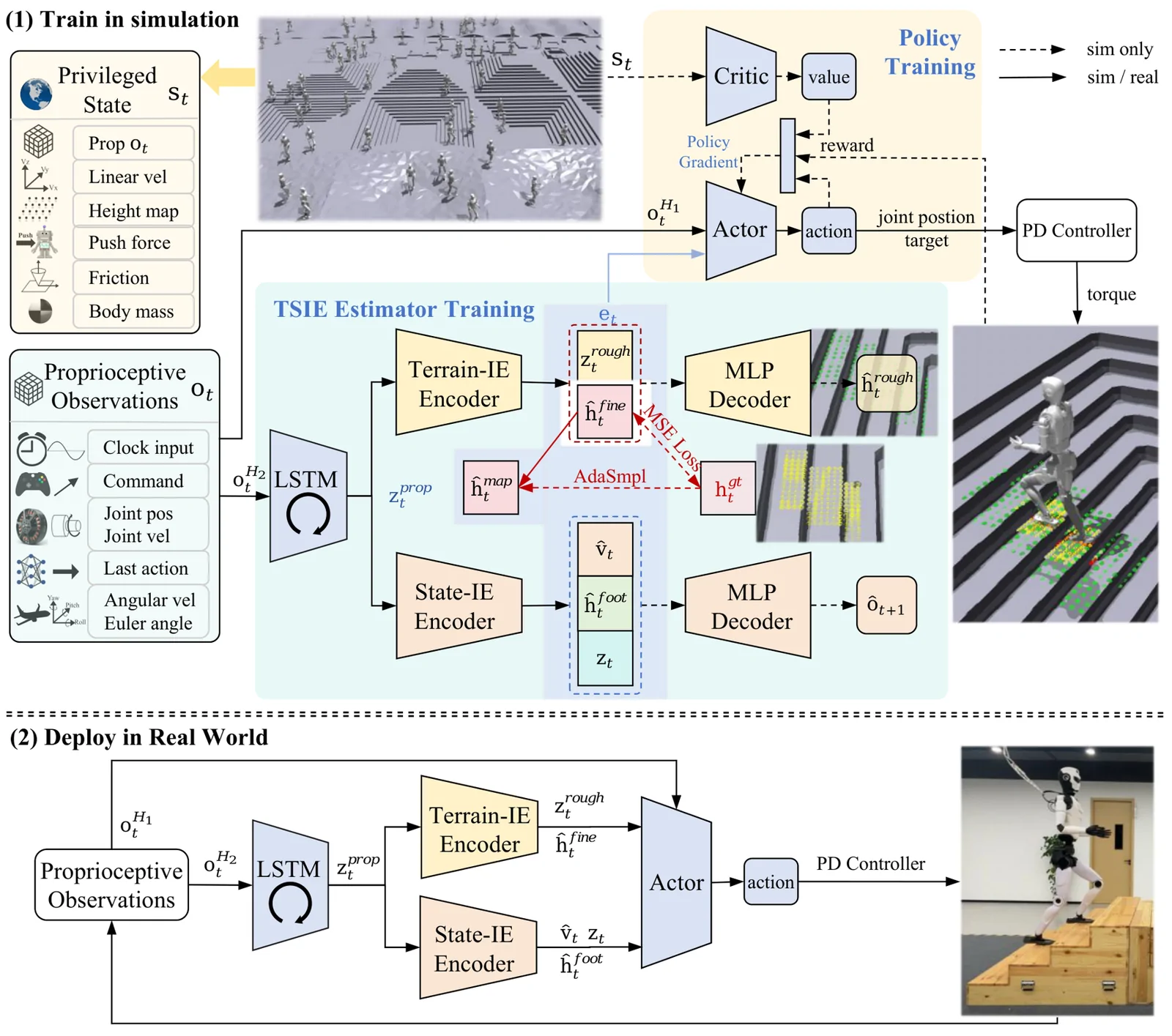
Overview of the TSIE framework. During simulation training (**top**), the LSTM history encoder, Terrain-IE, State-IE, Actor, and Critic are jointly optimized under an asymmetric Actor-Critic framework using privileged information, with AdaSmpl improving early-stage training stability. During real-world deployment (**bottom**), only proprioceptive observations are available, and the trained LSTM, Terrain-IE Encoder, State-IE Encoder, and Actor operate online to estimate multi-scale terrain and robot states for real-time control.

As shown in Figure 1, the TSIE framework consists of an Actor-Critic network and an estimator network. The upper part of the framework illustrates the privileged asymmetric reinforcement learning pipeline in simulation, while the lower part presents the real-world deployment pipeline. TSIE adopts an asymmetric Actor-Critic architecture, which simplifies the conventional two-stage training paradigm into a single-stage learning process. The policy network, i.e., the Actor, only receives proprioceptive observations available during deployment, while the Critic can access additional privileged information during training. The estimator network, including the LSTM history encoder, Terrain-IE, and State-IE, is jointly optimized with the policy during training, whereas only the trained LSTM, Terrain-IE Encoder, State-IE Encoder, and Actor are executed online during deployment. The optimization process contains two concurrent objectives: Actor-Critic policy optimization and estimator optimization. Specifically, proximal policy optimization (PPO) is jointly performed with supervised learning, and adaptive sampling (AdaSmpl) is introduced to reduce the exploration difficulty in the early training stage. The learned policy can be deployed on the real robot through zero-shot sim-to-real transfer.

#### 3.1.1. Policy Network

The input to the policy network consists of two parts: the robot proprioceptive observation $o_{t}\in R^{40}$ and the estimator output $e_{t}$. To enable the policy to rapidly respond to instantaneous foot–terrain contacts, we use a short history window with $H_{1}=5$, denoted as $o_{t}^{H_{1}}\in R^{40\times 5}$, to capture the robot’s short-term dynamic responses. The policy network takes $o_{t}^{H_{1}}$ and $e_{t}$ as inputs and directly generates the action $a_{t}$ for robot control. The proprioceptive observation $o_{t}$ is directly obtained from joint encoders and IMU, and is defined as

(1) $$o_{t}={\left[\begin{array}{ccccccc} p_{t} & c_{t} & q_{t} & {\dot{q}}_{t} & a_{t-1} & \theta_{t} & \omega_{t} \end{array}\right]}^{T},$$

where $p_{t}\in R^{2}$ is the clock input, $c_{t}\in R^{3}$ is the velocity command, $q_{t}\in R^{10}$ and ${\dot{q}}_{t}\in R^{10}$ are the joint positions and velocities, respectively, $a_{t-1}\in R^{10}$ is the previous action, $\theta_{t}\in R^{2}$ denotes the base Euler angles, and $\omega_{t}\in R^{3}$ denotes the base angular velocity.

Following prior work on gait-phase-based locomotion control [30], we define the clock input as $p_{t}=\left[\sin\left(2\pi \phi_{t,1}\right),\sin\left(2\pi \phi_{t,2}\right)\right]$ to guide the policy toward periodic gait generation. Here, $\phi_{t,i}$, i=1,2, are two time-varying phase variables corresponding to the two legs. They are updated according to the phase offset and gait period *T* as

(2) $$\begin{array}{cc} \phi_{t+1,1} & =\phi_{t,1}+dt/T,\\ \phi_{t+1,2} & =\phi_{t+1,1}+0.5, \end{array}$$

where dt is the discrete time step. During phase update, each $\phi_{i}$ is wrapped into the range [0,1] to generate a periodic phase signal. For the standing gait, the clock input is set to zero.

The estimator output $e_{t}$ represents the implicit-explicit estimation of privileged observations and is defined as

(3) $$e_{t}={\left[\begin{array}{ccccc} {\hat{v}}_{t} & {\hat{h}}_{t}^{foot} & z_{t} & {\hat{h}}_{t}^{fine} & z_{t}^{rough} \end{array}\right]}^{T}.$$

It consists of the explicitly estimated base linear velocity ${\hat{v}}_{t}\in R^{3}$, the explicitly estimated foot-centered height map ${\hat{h}}_{t}^{foot}\in R^{21\times 2}$, the purely implicit latent vector $z_{t}\in R^{16}$, the explicitly estimated local high-resolution height map ${\hat{h}}_{t}^{fine}\in R^{169}$, and the implicit representation of the global low-resolution height map $z_{t}^{rough}\in R^{32}$.

#### 3.1.2. Value Network

To improve the performance of both the policy and the estimator, the input to the value network, denoted as $s_{t}$, includes not only the proprioceptive observation $o_{t}$, but also privileged observations, including the base linear velocity $v_{t}\in R^{3}$, the local high-resolution height map $h_{t}^{fine}\in R^{169}$, the global low-resolution height map $h_{t}^{rough}\in R^{169}$, and other privileged information $p_{t}$. It is defined as

(4) $$s_{t}={\left[\begin{array}{ccccc} o_{t} & v_{t} & h_{t}^{fine} & h_{t}^{rough} & p_{t} \end{array}\right]}^{T}.$$

The detailed observation space is summarized in Table 1.

#### 3.1.3. Action Space

The policy outputs joint position offsets as the action vector $a_{t}\in R^{10}$. The desired joint position is computed as

(5) $$q_{target}=q_{0}+a_{t}*\alpha_{a},$$

where $q_{0}$ denotes the default joint position, and $\alpha_{a}$ is the action scaling factor. The desired joint position is then converted into torque commands by a low-level PD controller with fixed gains and zero desired joint velocity:

(6) $$\tau_{target}=k_{p}*\left(q_{target}-q_{t}\right)-k_{d}*{\dot{q}}_{t}.$$

The policy runs at 100 Hz, while the low-level PD controller runs at 1 kHz.

#### 3.1.4. Reward Function

Most reward terms follow common designs used in prior studies on legged locomotion [11,19,23], while their coefficients are tuned to guide the robot toward stable and efficient locomotion over different terrains and to avoid unsafe or unnatural motions. The detailed reward terms are listed in Table 2. For complex-terrain scenarios, we make necessary modifications to the foot-stance reward in (7) and the swing penalty in (8).

The desired stance phase is defined as $C\left(\phi_{t,i}\right)=\phi_{t,i}<\phi_{stance}$, where $\phi_{stance}$ determines the ratio between the stance and swing phases within a gait cycle. A larger $\phi_{stance}$ encourages a longer stance duration. In our implementation, we set $\phi_{stance}=0.65$ and the gait period to T=0.9. During the stance phase, the reward penalizes foot velocity, whereas during the swing phase, it penalizes foot contact force.

The decay coefficient λ is introduced to ensure that the periodic phase signal can guide the generation of periodic gaits at low speeds, preventing unnatural shuffling motions. Meanwhile, for medium and high speed tasks such as continuous stair ascent and descent, the gait-period constraint is relaxed to improve terrain adaptability and disturbance rejection. To ensure smooth reward modulation, cosine interpolation is adopted, as shown in (9).

(7) $$r_{\mathrm{contact}}=-\lambda \sum_{f=1}^{2}C\left(\phi_{t,i}\right)\left[1-\exp\left(\frac{∥v_{f,xy}∥}{5}\right)\right]$$

(8) $$r_{\mathrm{swing}}=-\lambda \sum_{f=1}^{2}\left[1-C(\phi_{t,i})\right]\left[1-\exp\left(\frac{∥F_{f}∥}{50}\right)\right]$$

(9) $$\lambda =\left\{\begin{array}{cc} 0.5(1+\cos(\pi \cdot |v_{x}^{\mathrm{cmd}}|/0.5)), & |v_{x}^{\mathrm{cmd}}|<0.5\\ 0, & |v_{x}^{\mathrm{cmd}}|>0.5 \end{array}\right.,$$

### 3.2. TSIE Estimator

#### 3.2.1. Long-Horizon Proprioceptive History Encoder

Previous studies have shown that, compared with single-frame proprioceptive observations, long-horizon proprioceptive observations can more effectively capture the dynamic interaction process between the robot and the environment, including foot-contact feedback, body motion responses, and terrain-induced disturbances [17,27]. Based on this observation, we design a Long-Horizon Proprioceptive History Encoder, which uses an LSTM module to extract implicit dynamic features from long-horizon historical observations generated by robot–environment interactions. These features provide a unified representation basis for the subsequent Terrain-IE and State-IE modules.

Specifically, the LSTM module takes a proprioceptive observation history of length $H_{2}=15$, denoted as $o_{t}^{H_{2}}\in R^{40\times 15}$, and outputs an implicit feature vector $z_{t}^{\mathrm{prop}}\in R^{64}$. This vector encodes historical information related to the robot motion state, terrain friction, and terrain geometry. In addition, compared with methods that rely only on the current observation, the long-horizon history encoding provides stronger temporal modeling and noise suppression capabilities. It can mitigate the effects of stochastic sensor noise and transient disturbances, thereby improving the accuracy, stability, and environmental adaptability of subsequent terrain and state estimation.

#### 3.2.2. Terrain Implicit-Explicit Estimator

Traditional proprioception-based terrain estimation methods usually rely on single-scale implicit terrain representations to avoid failures caused by explicit estimation errors. However, such representations cannot effectively capture both global terrain trends and local geometric details, which limits policy performance over complex terrains.To address this issue, we propose Terrain-IE, a terrain implicit-explicit estimator that jointly estimates local high-resolution and global low-resolution height maps. Given the implicit feature vector $z_{t}^{prop}$ from the long-horizon proprioceptive history encoder, Terrain-IE uses an encoder-decoder architecture to estimate two-scale terrain representations in an implicit-explicit manner. The estimated terrain features are then provided to the policy network for terrain perception and locomotion control.

Specifically, Terrain-IE first explicitly estimates a local high-resolution height map ${\hat{h}}_{t}^{fine}\in R^{169}$, supervised by the ground-truth local high-resolution height map $h_{t}^{fine}$. This height map covers a 0.3m×0.3m square region around the robot with a spatial resolution of 5cm. It captures local geometric details, such as stair edges and obstacle heights, thereby improving foothold planning accuracy and locomotion stability. The explicit local high-resolution height map estimation is optimized using the Mean Squared Error (MSE) loss, defined as

(10) $$L_{\mathrm{fine}}=\mathrm{MSE}\left({\hat{h}}_{t}^{fine},h_{t}^{fine}\right).$$

Meanwhile, Terrain-IE encodes an implicit latent vector $z_{t}^{rough}\in R^{32}$ for the global low-resolution height map to represent large-scale terrain trends. The latent vector $z_{t}^{rough}$ is then concatenated with the explicitly estimated local high-resolution height map ${\hat{h}}_{t}^{fine}$ and decoded into the estimated global low-resolution height map ${\hat{h}}_{t}^{rough}\in R^{169}$. The corresponding supervision signal is the ground-truth global low-resolution height map $h_{t}^{rough}$, which covers a 0.6m×0.6m square region around the robot with a spatial resolution of 10cm. Compared with local explicit height map estimation, global implicit height map reconstruction focuses more on large-scale terrain undulations and overall terrain variations, thereby providing more anticipatory terrain perception for gait adaptation. The reconstruction loss is defined as

(11) $$L_{\mathrm{rough}}=\mathrm{MSE}\left({\hat{h}}_{t}^{rough},h_{t}^{rough}\right).$$

The local high-resolution height map provides rich geometric details, but its high dimensionality may cause accumulated estimation errors when it is optimized only with local supervision, which can further affect policy learning stability. In contrast, the global low-resolution height map, although coarser, preserves important terrain structure variations. Therefore, by preserving the gradient connection during the extraction of $z_{t}^{rough}$, the global reconstruction loss $L_{\mathrm{rough}}$ provides additional geometric constraints for local high-resolution height map estimation, improving the accuracy of explicit terrain estimation.The resulting multi-scale implicit-explicit estimation mechanism captures both local geometric details and global terrain trends, thereby enhancing the policy’s adaptability to complex unstructured terrains. To further improve the robustness of the implicit terrain representation, an $L_{1}$ regularization term is imposed on $z_{t}^{rough}$. The overall training loss of Terrain-IE is defined as

(12) $$L_{\mathrm{TIE}}=L_{\mathrm{fine}}+L_{\mathrm{rough}}+{∥z_{t}^{rough}∥}_{1}.$$

#### 3.2.3. State Implicit-Explicit Estimator

Relying solely on the multi-scale terrain height maps estimated by Terrain-IE is insufficient for policy learning. Prior studies have shown that robots can implicitly infer their own states and surrounding environment by predicting future proprioceptive observations. Motivated by this idea, we design State-IE as the state estimation branch of the TSIE estimator.State-IE is trained in parallel with Terrain-IE and also takes the low-dimensional implicit feature vector $z_{t}^{prop}$ from the LSTM module as input. It employs an encoder–decoder structure to perform implicit-explicit estimation of the base linear velocity $v_{t}$, the foot-centered height map $h_{t}^{foot}$, and the future proprioceptive observation $o_{t+1}$, thereby improving robustness to unknown dynamic disturbances. The foot-centered height map is defined on a fixed two-dimensional sampling grid attached to each foot and moving together with it. The sampling locations are computed in the world frame, where the corresponding terrain heights are directly queried from the simulator as supervision during training. The explicitly estimated base linear velocity ${\hat{v}}_{t}$ and foot-centered height map ${\hat{h}}_{t}^{foot}$ can significantly improve velocity tracking performance during training. Meanwhile, the foot-centered height map provides terrain information around the feet, which helps foothold planning and reduces unsafe foot collisions. We optimize these explicit estimates using the mean squared error (MSE) between the estimated values and the corresponding ground-truth values. The estimation loss is defined as

(13) $$L_{\mathrm{est}}=\mathrm{MSE}\left({\hat{v}}_{t},v_{t}\right)+\mathrm{MSE}\left({\hat{h}}_{t}^{\mathrm{foot}},h_{t}^{\mathrm{foot}}\right).$$

Following prior work [17], we adopt a variational autoencoder (VAE) structure to extract the implicit latent vector $z_{t}$. When decoded together with the estimated linear velocity and foot-centered height map, this latent vector reconstructs ${\hat{o}}_{t+1}$ to predict the future proprioceptive observation $o_{t+1}$. This reconstruction encourages the latent representation to implicitly capture dynamic parameters and environmental properties, such as ground friction, external pushes, payload variations, and terrain obstacles, while suppressing sensor noise in proprioceptive observations.

The VAE loss consists of a reconstruction loss based on MSE and a latent regularization term based on the Kullback–Leibler (KL) divergence:

(14) $$L_{\mathrm{VAE}}=\mathrm{MSE}\left({\hat{o}}_{t+1},o_{t+1}\right)+D_{\mathrm{KL}}\left(q\left(z_{t}|z_{t}^{prop}\right)∥p(z_{t})\right).$$

Here, $q\left(z_{t}|z_{t}^{prop}\right)$ denotes the posterior distribution of $z_{t}$, and $p(z_{t})$ denotes the prior distribution, parameterized as a standard normal distribution.

#### 3.2.4. Adaptive Sampling

During the early stage of training, Terrain-IE has not yet fully converged, and its explicitly estimated terrain output may contain large noise and errors, making it difficult to accurately represent the true terrain structure. Directly feeding the estimated terrain into the policy network may interfere with policy learning and reduce training stability. To address this issue, we introduce an adaptive sampling method. During policy training, the ground-truth terrain height map $h_{t}^{fine}$ is used to replace the estimated terrain ${\hat{h}}_{t}^{fine}$ as the policy input with probability $p_{\mathrm{smpl}}$.

The probability is adaptively determined by the coefficient of variation (CV) of the returns, defined as

(15) $$\mathrm{CV}\left(R\right)=\frac{\sigma (R)}{\mu (R)},$$

where $R\in R^{m\times 1}$ denotes the accumulated returns from *m* parallel environments, and σ(·) and μ(·) denote the standard deviation and mean, respectively. The CV of returns is adopted because it jointly characterizes the mean and variability of policy returns, providing a scale-invariant measure of the policy convergence status. To enable a smooth transition from ground-truth terrain to estimated terrain, the hyperbolic tangent (*tanh*) function is employed to map the CV to the sampling probability. Compared with a linear mapping, the *tanh* function provides a smooth, bounded, and asymptotically saturating transformation, preventing abrupt changes in the sampling probability and thereby mitigating policy optimization instability caused by sudden shifts in the observation distribution. Based on the coefficient of variation, the probability of adopting the ground-truth terrain is defined as

(16) $$p_{\mathrm{smpl}}=\tanh\left(\mathrm{CV}\left(R\right)\right).$$

At the early stage of training, when the policy has not yet converged, the return variance across different environments is large, resulting in a higher CV(R) and thus a larger $p_{\mathrm{smpl}}$. In this case, the policy is trained more frequently with ground-truth terrain inputs, which helps the robot learn critical terrain features and reduces exploration difficulty. As training progresses, the return variance gradually decreases, and $p_{\mathrm{smpl}}$ is reduced accordingly. The policy then gradually transitions to using the terrain estimated by Terrain-IE, thereby improving its robustness to estimation errors and noise. During real-world deployment, the policy uses only the estimated terrain as input.

### 3.3. Training Details

#### 3.3.1. Simulation Parameters

To train a robust locomotion policy efficiently, we construct a large-scale parallel reinforcement learning environment based on NVIDIA Isaac Gym. A total of 4096 environments are simulated in parallel, and an NVIDIA RTX 4090 GPU is used to accelerate both physics simulation and network training, including the Actor, Critic, and estimator networks.

The maximum episode duration is set to 24s. The policy operates at a control frequency of 100Hz, while the physics simulation runs at 200Hz. All joints are controlled by low-level PD controllers. For the knee joints, the proportional and derivative gains are set to 150 and 4, respectively. To mitigate foot–ground impact, lower gains of 30 and 1 are used for the ankle joints, while the remaining joints use gains of 100 and 2.

The architectures of all network modules in the proposed TSIE framework are summarized in Table 3, and the PPO hyperparameters are listed in Table 4.

#### 3.3.2. Training Curriculum

**Figure 2:**
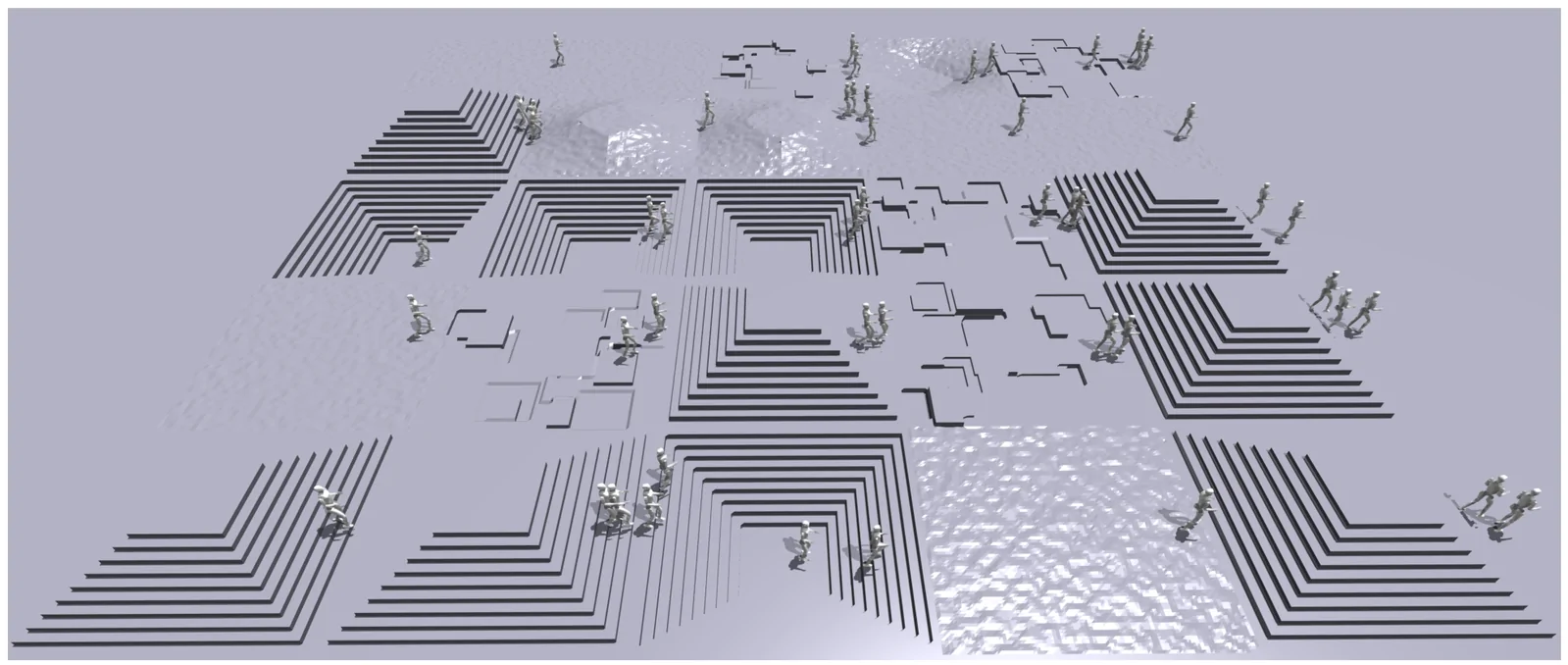
Terrain types used for simulation training.

To improve the terrain adaptability and generalization capability of the policy in real-world environments, we design five representative terrain types in simulation, including flat ground, rough terrain, slopes, stairs, and discrete obstacles, as shown in Figure 2. Following the principle of curriculum learning adopted in prior work, the terrain difficulty is progressively increased during training, allowing the policy to gradually adapt to more challenging environments. This strategy effectively alleviates convergence instability in the early training stage and improves the generalization performance of the policy across different terrains.

For velocity-command tracking, the forward velocity command is sampled from the range of [−0.8,1.0]m/s, the lateral velocity command is sampled from [−0.8,0.8]m/s, and the yaw angular velocity command is sampled from [−1.2,1.2]rad/s.

#### 3.3.3. Domain Randomization

To improve the robustness of the policy against sensor noise, actuator model mismatch, and environmental uncertainties, thereby facilitating smooth sim-to-real transfer, we randomize the observation noise, payload, center-of-mass position, external pushes, ground friction, actuator latency, motor stiffness, motor damping, and motor strength during training. The randomized physical parameters and their corresponding ranges are detailed in Table 5.

## 4. Experiments

To evaluate the effectiveness of the proposed TSIE framework, we conduct experiments in both simulation environments and on a real robot platform. This section first introduces the experimental setup, followed by the presentation and analysis of the experimental results. The experiments are designed to answer the following four questions: (i) Does the proposed framework improve terrain traversal performance compared with its ablation variants and baseline methods? (ii) Can Terrain-IE accurately estimate terrain height maps, and does the proposed multi-scale terrain estimation improve terrain estimation accuracy? (iii) Does State-IE improve velocity-tracking performance across different terrains, and can it implicitly infer surrounding environmental information? (iv) How well does TSIE achieve zero-shot sim-to-real transfer?

### 4.1. Experimental Setup

**Figure 3:**
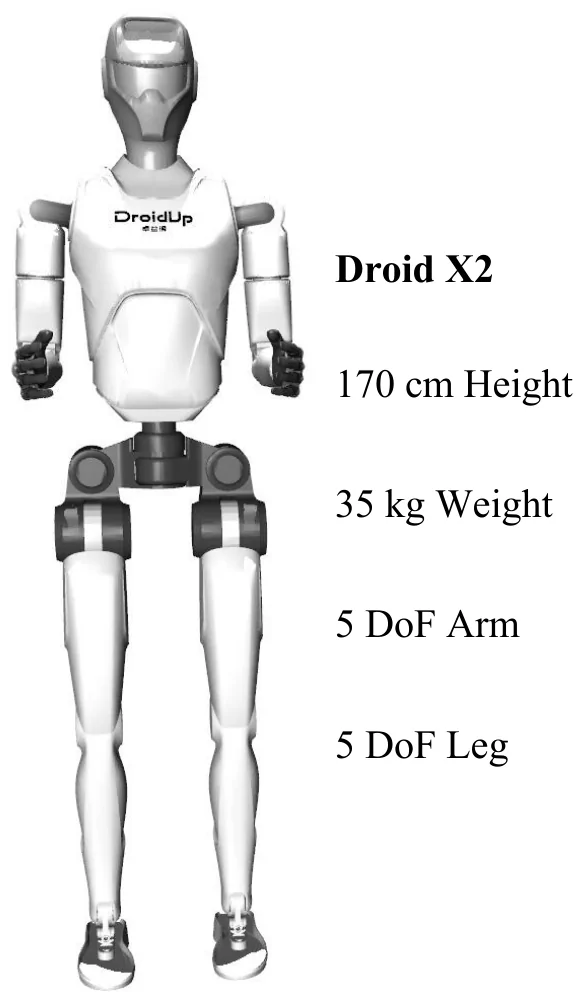
Hardware configuration of the Droid X2 humanoid robot, including its physical parameters and degrees of freedom.

All simulation and real-world experiments are conducted on the full-sized humanoid robot Droid X2. As shown in Figure 3, the robot has a mass of 35kg, a height of 1.7m, and a peak joint torque of 70N·m. It has 20 actuated degrees of freedom, including five degrees of freedom in each leg and five in each arm. For the purpose of this study, we focus on leg control, while the arm motors are kept at their default positions.

Unlike conventional robots with quasi-direct-drive joints, Droid X2 adopts a tendon-driven actuation scheme to reduce leg mass, inertia, and energy consumption. Three quasi-direct-drive motors integrated in the hip module jointly actuate the hip, knee, and ankle joints through chains and tendons, which significantly increases the complexity of real-world control. To deploy the learned policy on the physical robot, we map joint torques to motor torques using a static Jacobian. The low-level joint PD controller runs at 1kHz. To evaluate the practical deployability of the proposed method, the trained TSIE model was converted into the ONNX format and deployed on an onboard RK3588 embedded edge-computing platform. The deployed model contains approximately 0.92 million parameters. At a control frequency of 100 Hz, the average inference latency is approximately 3.62 ms per control cycle, which is significantly lower than the 10 ms control period. These results demonstrate that TSIE satisfies the real-time computational requirements of bipedal robot control.

To comprehensively evaluate the effectiveness of TSIE and the contribution of its core components, we conduct extensive simulation and real-world experiments, including four ablation studies and comparisons with two baseline methods:

- TSIE: The complete proposed method, which includes long-horizon proprioceptive history encoder, the Terrain-IE module, and the State-IE module.
- TSIE w/o Long History Obs: This variant removes the long-horizon proprioceptive history input and uses only single-frame observations for terrain and state estimation, while keeping the remaining framework unchanged. It is used to evaluate the contribution of long-horizon historical information to environmental perception.
- TSIE w/o State-IE: This variant removes the State-IE module to evaluate the influence of state estimation and future-state prediction on policy performance.
- TSIE w/o reconstructing ${\hat{h}}_{t}^{rough}$: This variant removes the reconstruction of the global low-resolution height map ${\hat{h}}_{t}^{rough}$ in Terrain-IE and only uses the local high-resolution height map estimation. It is used to verify the importance of reconstructing global terrain trends.
- TSIE w/o estimating ${\hat{h}}_{t}^{fine}$: This variant removes the explicit estimation of the local high-resolution height map ${\hat{h}}_{t}^{fine}$ and only retains the reconstruction of the global low-resolution height map. It is used to verify the importance of perceiving local terrain details.
- DWL [19]: A learning-based implicit privileged state estimation method that employs a Denoising World Model to learn implicit representations of privileged information, including terrain and robot dynamics, enabling end-to-end humanoid locomotion over complex terrains.
- VPIES [18]: A learning-based privileged-information encoding and state estimation method that leverages a Variational Privileged Information Encoder together with a dual-timescale proprioceptive history encoder to learn implicit terrain representations while explicitly estimating the robot’s linear velocity for policy learning.

### 4.2. Simulation Experiments

#### 4.2.1. Framework Comparison

**Figure 4:**
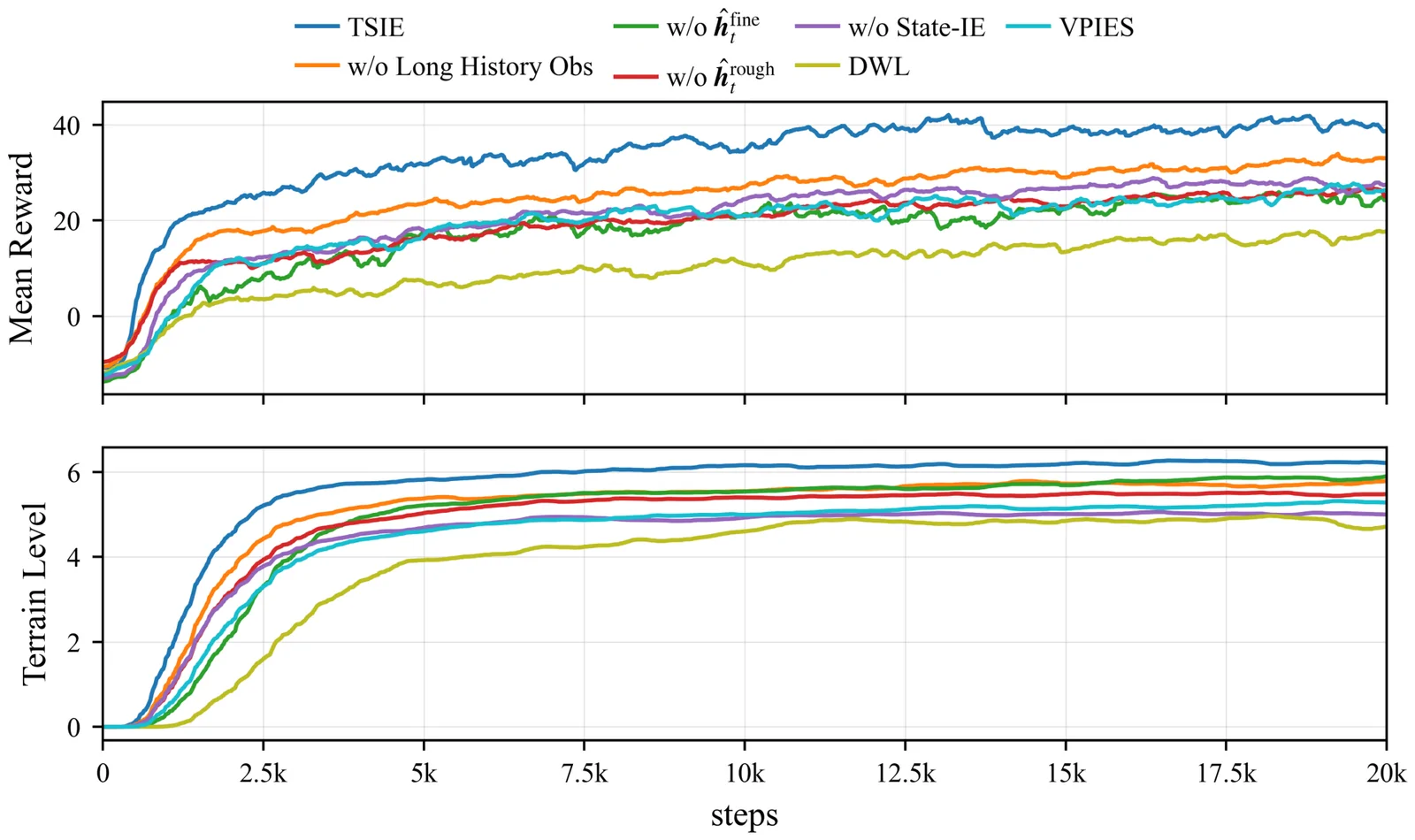
Comparison of the average reward and terrain curriculum level of different methods during training. The upper plot shows the average reward, while the lower plot shows the progression of the terrain curriculum level.

To analyze the performance evolution of different methods during training, Figure 4 shows the average reward and terrain curriculum level of the seven methods. For a fair comparison, all methods use the same reward function and are trained for 20,000 iterations.

Overall, TSIE achieves the best performance in terms of both training efficiency and final convergence. DWL reconstructs all privileged information through a unified decoder and lacks explicit estimation of key quantities such as height maps and base linear velocity. As a result, it obtains the lowest average reward and terrain curriculum level. VPIES introduces a dual-timescale history encoder and improves training performance compared with DWL. However, its terrain information is mainly inferred from implicit features without explicit geometric constraints, leading to lower final performance than the complete TSIE. In contrast, TSIE exhibits faster reward growth and curriculum progression in the early training stage, and surpasses the final convergence levels of the other methods after approximately 5000 iterations. This indicates that the proposed framework can effectively improve policy learning efficiency.

Further analysis of the ablation results shows that the two single-scale terrain estimation variants both lead to lower exploration efficiency and final curriculum levels. Specifically, TSIE w/o ${\hat{h}}_{t}^{fine}$ only relies on global implicit height map reconstruction and fails to capture local geometric details, resulting in slower curriculum progression. TSIE w/o ${\hat{h}}_{t}^{rough}$ only relies on local explicit height map estimation and lacks the capability to model global terrain trends, which limits its performance improvement on high-difficulty terrains. These results demonstrate the complementarity between local explicit estimation and global implicit reconstruction, and verify the importance of multi-scale terrain implicit-explicit estimation for complex-terrain learning.

In addition, removing either long-horizon proprioceptive observations or State-IE leads to slower reward growth and lower final terrain curriculum levels. This indicates that historical observations and state implicit-explicit estimation play important roles in environmental perception, state modeling, and adaptation to dynamic disturbances over complex terrains.

To evaluate the terrain traversal capability and locomotion robustness of different methods, each policy is tested in 800 parallel simulation environments. The test scenarios include stairs, slopes, and discrete obstacles with different difficulty levels, resulting in eight terrain settings in total, with 100 trials conducted for each terrain. A trial is considered successful if the robot traverses the entire terrain stably without falling, obvious instability, or premature termination.

The traversal success rates on complex terrains are reported in Table 6. TSIE achieves the highest success rate across all test terrains, with more pronounced advantages on high-difficulty stairs and discrete obstacles. For example, TSIE achieves success rates of 94% and 93% on 20cm stairs and 20cm discrete obstacles, respectively, which are significantly higher than those of DWL, 72% and 68%, and VPIES, 89% and 86%. This result is consistent with the training curves, indicating that the dual-branch terrain–state implicit-explicit estimation framework not only improves training efficiency but also enhances complex-terrain traversal capability.

The ablation results show that multi-scale terrain modeling is particularly important for high-difficulty terrains. In low and medium difficulty terrains, the performance gap between the single-scale variants and the complete TSIE is relatively small. However, on 20cm stairs and discrete obstacles, the success rates decrease to 90% and 89% when only global implicit reconstruction is retained, and further decrease to 85% and 83% when only local explicit estimation is retained. These results indicate that local explicit estimation and global implicit reconstruction are complementary, providing fine-grained geometric information and anticipatory terrain perception, respectively. In addition, removing either the long-horizon history encoder or State-IE leads to a more significant performance degradation on high-difficulty terrains, with the success rate on 20cm stairs decreasing to 83% and 80%, respectively. This demonstrates that long-horizon proprioceptive observations, state estimation, and future-state prediction are critical for environmental perception and dynamic modeling over complex terrains. They enhance the policy’s adaptability to partially observable environments, dynamic disturbances, and complex contact conditions, thereby improving locomotion robustness and generalization on challenging unstructured terrains.

Table 7 further reports the number of failures of different methods during stair ascent and descent. For each stair difficulty level, ascent and descent failures are evaluated separately over 1000 stair environments. The results show that the number of failures increases for all methods as the stair height increases, with a more pronounced increase during stair descent. This indicates that stair descent imposes higher requirements on dynamic balance control and terrain perception than stair ascent.Compared with all baseline methods and ablation variants, the complete TSIE framework consistently achieves the lowest number of failures across all stair difficulty levels, with more significant advantages in high-difficulty stair scenarios.

#### 4.2.2. Terrain-IE Estimation Analysis

To evaluate the terrain modeling capability of Terrain-IE, we analyze its performance from two aspects: training loss convergence and terrain estimation accuracy. Since AdaSmpl directly acts on the local high-resolution explicit height map estimation branch and affects how the estimated height map is used during policy training, we additionally introduce the w/o AdaSmpl variant to evaluate its influence on estimation stability and final accuracy. Specifically, we compare TSIE, w/o ${\tilde{h}}_{t}^{fine}$, w/o ${\tilde{h}}_{t}^{rough}$, and w/o AdaSmpl in terms of the local explicit height map estimation loss $L_{fine}$ and the global implicit height map reconstruction loss $L_{rough}$ during training. Here, $L_{fine}$ measures the estimation error between the estimated local high-resolution height map ${\tilde{h}}_{t}^{fine}$ and the ground-truth height map $h_{t}^{fine}$, while $L_{rough}$ measures the reconstruction error between the estimated global low-resolution height map ${\tilde{h}}_{t}^{rough}$ and the ground-truth height map $h_{t}^{rough}$.

**Figure 5:**
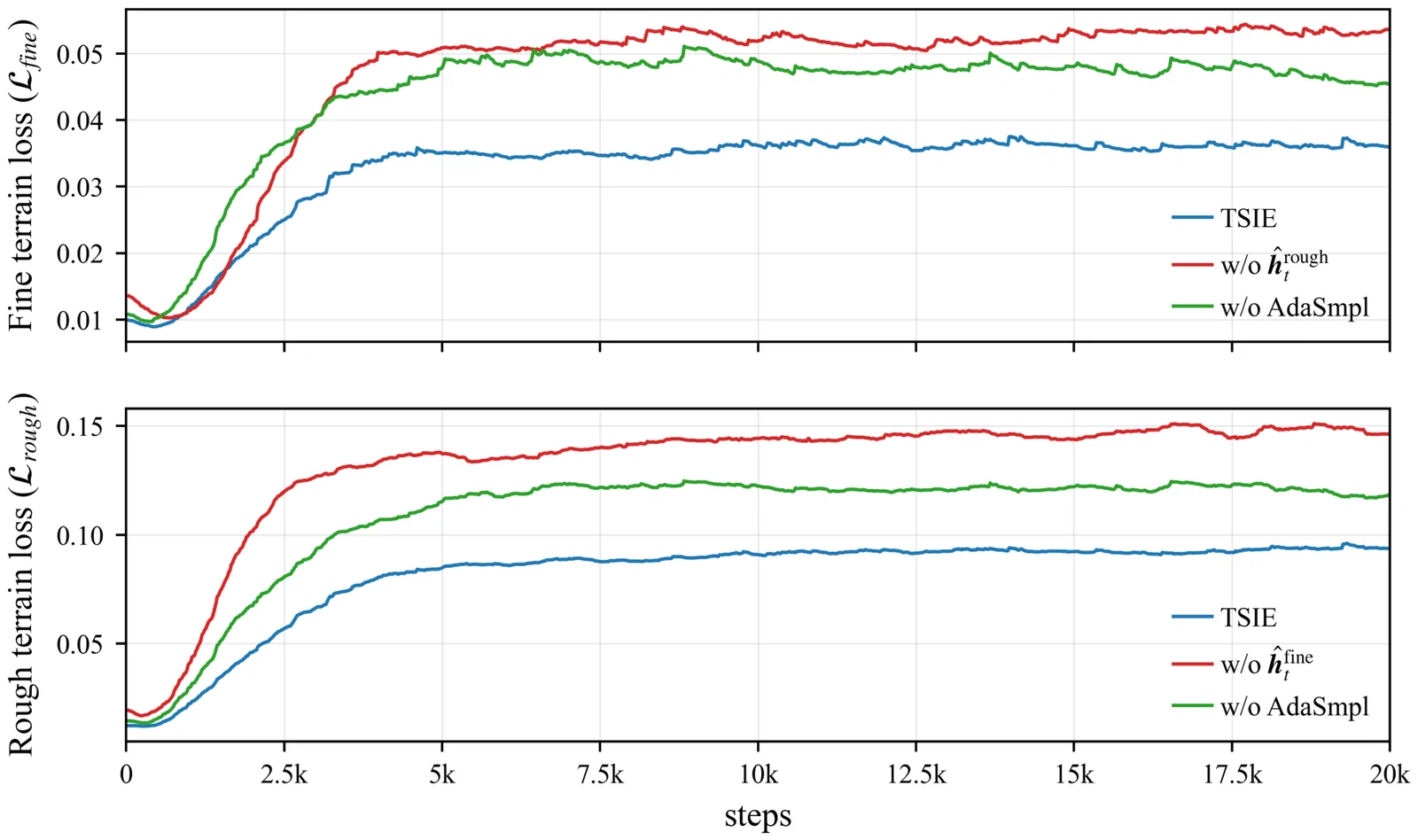
Comparison of the local high-resolution terrain estimation loss and the global low-resolution terrain reconstruction loss.

The results are shown in Figure 5. The complete TSIE achieves the lowest final errors for both losses and shows more stable convergence, indicating that local explicit height map estimation and global implicit height map reconstruction are complementary. When global implicit reconstruction is removed, $L_{fine}$ increases clearly, suggesting that the absence of global terrain-trend constraints weakens the stability of local high-resolution height map estimation. When local explicit estimation is removed, $L_{rough}$ increases significantly, indicating that relying only on global implicit features is insufficient to accurately recover local geometric details such as stair edges and obstacle heights.In addition, the w/o AdaSmpl variant produces higher errors than the complete TSIE for both losses. This indicates that directly using immature estimated terrain in the early training stage may interfere with policy learning and terrain modeling. By adaptively adjusting the use of ground-truth and estimated terrain, AdaSmpl effectively mitigates the instability caused by early estimation errors, thereby improving the training stability and final estimation accuracy of Terrain-IE.

**Figure 6:**
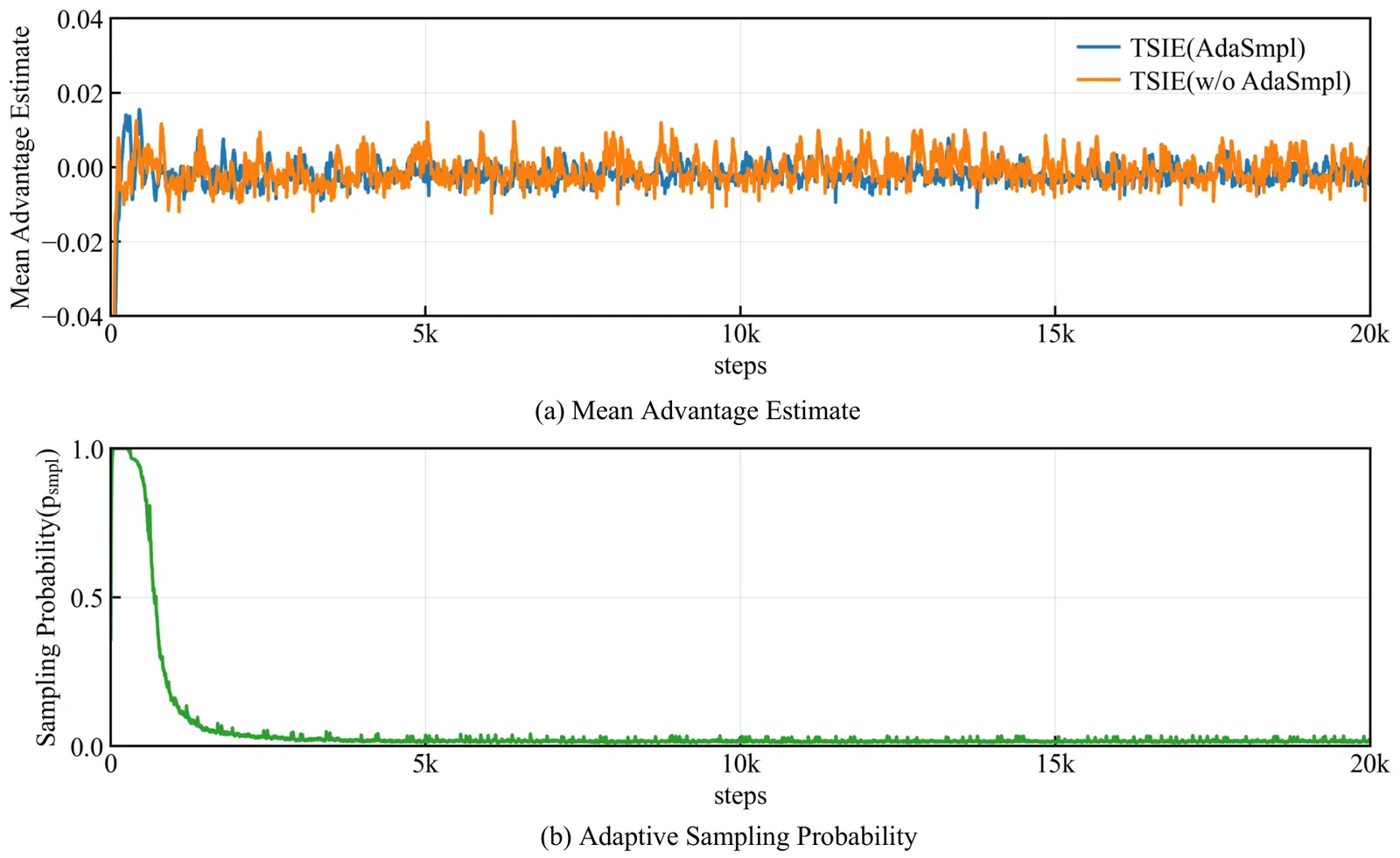
Analysis of the adaptive sampling mechanism. (**a**) Mean advantage estimate during training for TSIE and its ablation without AdaSmpl. (**b**) Adaptive sampling probability throughout training. The stable advantage estimation indicates that the gradual reduction of the sampling probability does not introduce noticeable instability into PPO optimization.

To verify that the adaptive sampling mechanism does not destabilize PPO optimization due to the gradual change in observation distribution, we further analyze the mean advantage estimate and the adaptive sampling probability during training, as shown in Figure 6. At the early stage of training, a high sampling probability allows the policy to learn primarily from ground-truth terrain representations, thereby reducing the exploration difficulty. As training progresses, the sampling probability gradually decreases, enabling a smooth transition toward estimated terrain representations. Throughout the entire training process, the mean advantage estimate of TSIE remains stable and exhibits no noticeable oscillation, divergence, or abrupt fluctuation compared with the variant without AdaSmpl. These results indicate that the proposed AdaSmpl achieves a smooth transition between ground-truth and estimated terrain representations without introducing instability or policy collapse during PPO optimization.These findings are also consistent with the stable training behavior and superior locomotion performance demonstrated by TSIE in the preceding experiments.

**Figure 7:**
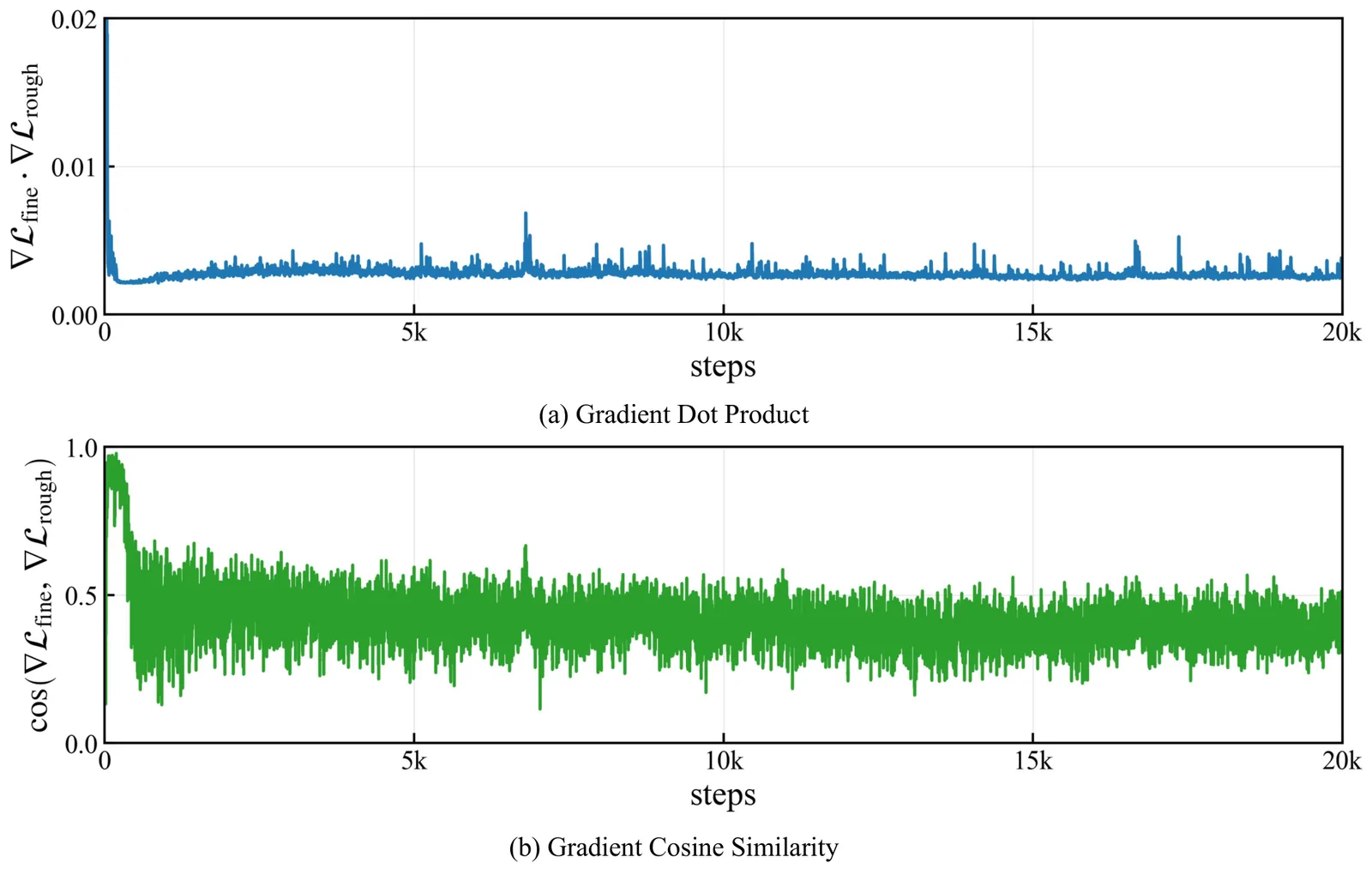
Gradient consistency analysis between the fine terrain estimation loss and the rough terrain reconstruction loss during training. (**a**) Gradient dot product. (**b**) Gradient cosine similarity. The consistently positive dot product and cosine similarity indicate that the two optimization objectives remain well aligned throughout training without noticeable gradient interference.

To verify that the proposed multi-scale optimization does not suffer from gradient interference, we further analyze the gradient dot product and cosine similarity between the fine-scale terrain estimation loss $L_{\mathrm{fine}}$ and the rough-scale terrain reconstruction loss $L_{\mathrm{rough}}$ throughout training, as shown in Figure 7. At the early stage of training, the cosine similarity is close to 1, indicating that the two objectives are highly aligned while jointly learning the fundamental terrain representations. As training progresses, the cosine similarity gradually stabilizes between 0.4 and 0.5, while the gradient dot product remains consistently positive. These results indicate that the gradients of the two objectives remain acute throughout training without noticeable destructive interference. Therefore, the global rough-scale terrain reconstruction loss provides effective guidance and regularization for the fine-scale terrain estimation during joint optimization.

To further quantitatively evaluate the terrain estimation accuracy of different methods, we compute the average height estimation error in cm on different terrains. The test scenarios include uphill slopes, downhill slopes, stair ascent, and stair descent at the highest difficulty level, and the results are reported in Table 8. TSIE achieves the lowest average estimation error in all test scenarios, with particularly clear advantages in stair scenarios. When global implicit terrain reconstruction is removed, the estimation error increases clearly across all scenarios, especially on stairs. This indicates that modeling global terrain trends helps recover the overall geometric structure of complex terrains. Removing AdaSmpl also leads to higher estimation errors, suggesting that the adaptive terrain adoption mechanism can mitigate the interference caused by early-stage estimation errors during policy learning and terrain modeling, thereby improving the stability and final accuracy of terrain estimation.

**Figure 8:**
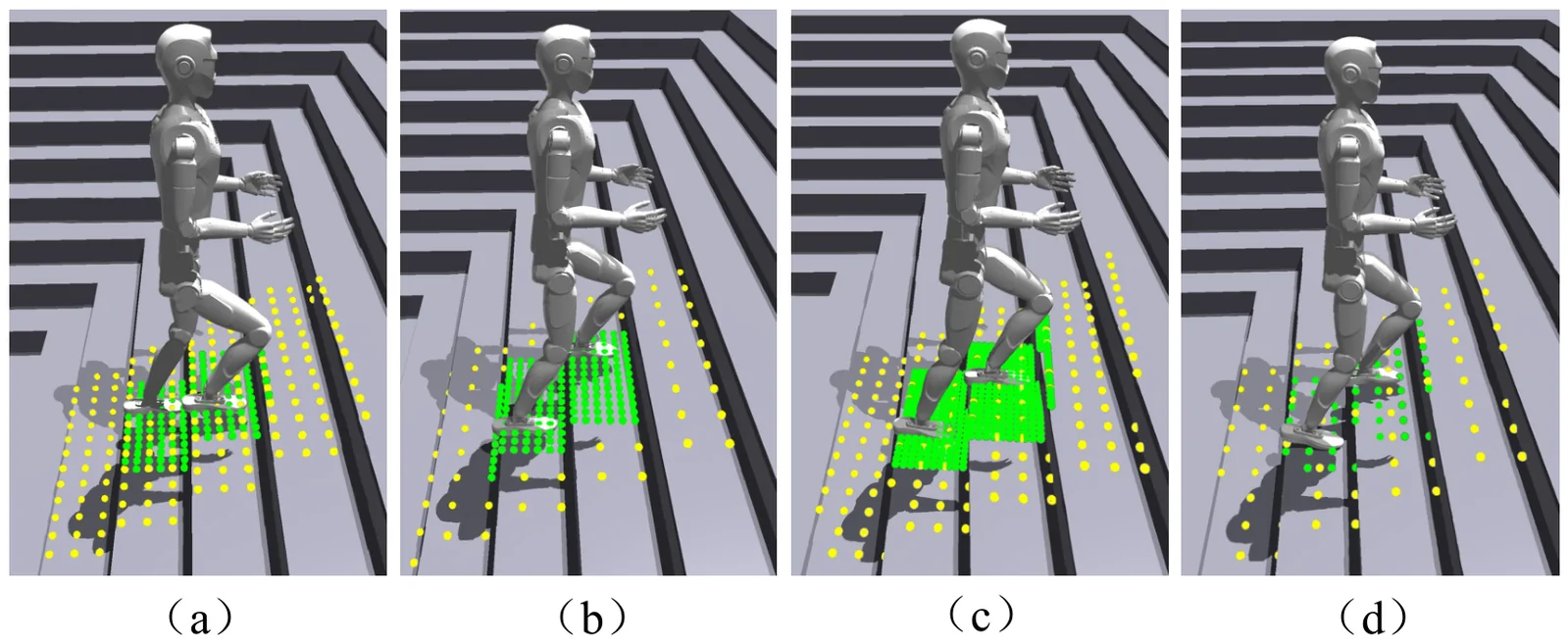
Visualization of the local/global height-map resolution configurations used in the resolution sensitivity analysis. (**a**) TSIE(5,10), (**b**) TSIE(5,15), (**c**) TSIE(3,10), and (**d**) TSIE(10,15), where the values denote the spatial resolutions (cm) of the local and global height maps, respectively.

To analyze the sensitivity of terrain estimation performance to the spatial resolutions of the height maps, we evaluated four different resolution configurations while keeping the physical coverage areas of the local and global height maps unchanged, as illustrated in Figure 8. The configurations are denoted as TSIE(5,10), TSIE(5,15), TSIE(3,10), and TSIE(10,15), where the two values represent the spatial resolutions (cm) of the local and global height maps, respectively. All configurations were evaluated on a 20cm stair terrain using 1000 parallel simulation trials. For each configuration, the stair traversal success rate was recorded, and MAE of the estimated height map was computed with respect to the ground-truth terrain height map.As shown in Table 9, reducing the height-map resolution significantly increases the terrain estimation error and degrades locomotion performance. When the global resolution is reduced from 10cm to 15cm, the MAE increases from 5.115cm to 6.039cm, while the success rate decreases from 0.945 to 0.912, indicating that an excessively coarse global resolution is insufficient to capture large-scale terrain variations. When both the local and global resolutions are further reduced to 10cm and 15cm, respectively, the MAE increases to 7.423cm and the success rate drops to 0.896, demonstrating that a coarse local resolution cannot accurately recover critical geometric structures such as stair edges. In contrast, increasing the local resolution from 5cm to 3cm only marginally reduces the MAE from 5.115cm to 5.107cm, while the success rate slightly decreases to 0.941. This suggests that a local resolution of 5cm is already sufficient to capture the geometric details around the foothold, and further increasing the resolution provides only marginal benefits. Therefore, the adopted local/global resolution configuration of 5cm/10cm achieves a favorable balance between local geometric detail representation, global terrain trend modeling, and locomotion performance.

**Figure 9:**
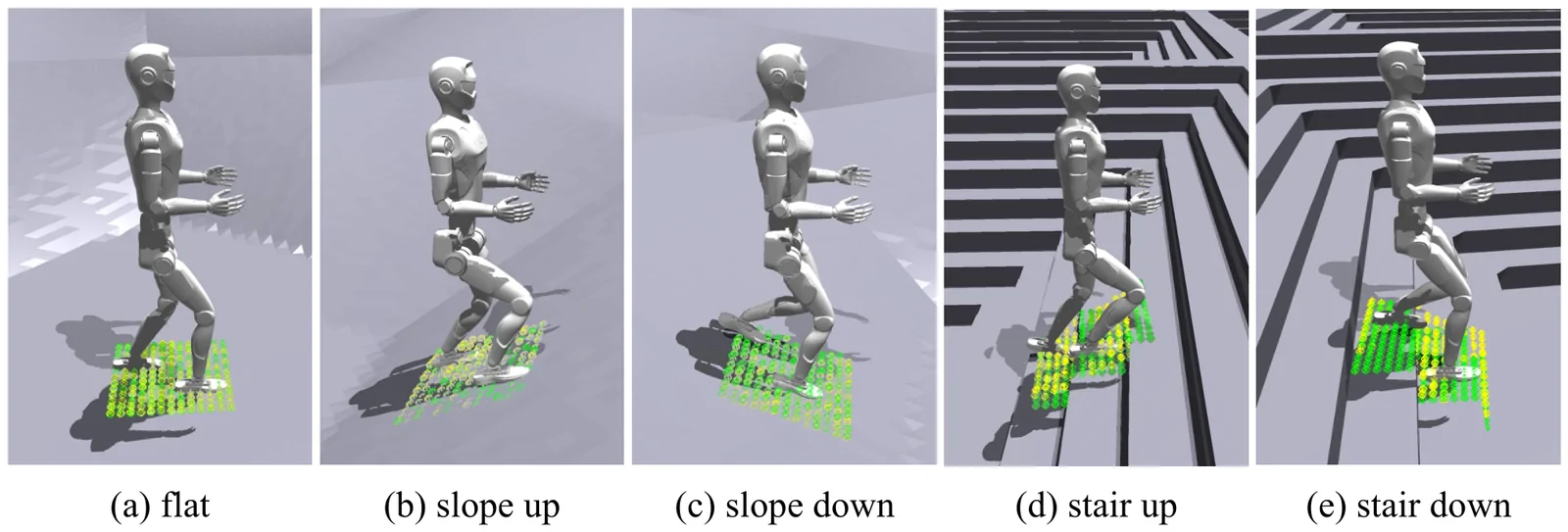
Visualization of terrain height map estimation results produced by TSIE.

Figure 9 visualizes the height map estimation results of TSIE under different terrain scenarios. The green points denote the ground-truth terrain heights, while the yellow points denote the terrain heights estimated by TSIE. The visualization results show that, without access to exteroceptive perception, TSIE can accurately recover key geometric structures of different terrains, including flat regions on level ground, continuous height variations on slopes, and local height discontinuities and stair-edge information on stairs.

Overall, the above results demonstrate that the local explicit estimation, global implicit reconstruction, and AdaSmpl mechanism in Terrain-IE jointly improve the accuracy, stability, and generalization capability of terrain height-map estimation, providing a more reliable multi-scale terrain representation for locomotion control over complex terrains.

#### 4.2.3. State-IE Estimation Analysis

**Figure 10:**
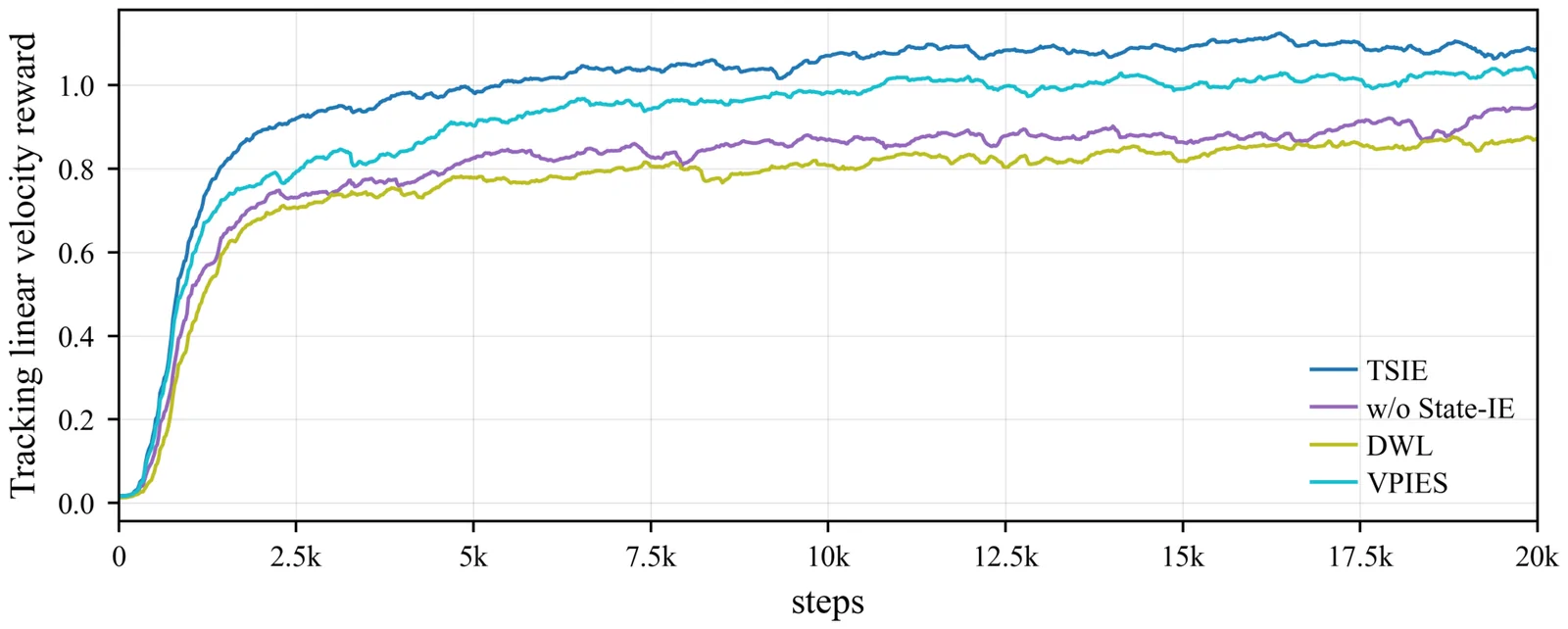
Comparison of the average linear velocity tracking reward of different methods during training.

To analyze the effect of State-IE on state estimation and velocity tracking, we compare TSIE, w/o State-IE, DWL, and VPIES, which adopt different linear velocity modeling strategies. This comparison is used to verify the effectiveness of State-IE in motion-state modeling. Figure 10 shows the average linear velocity tracking reward of different methods during training. TSIE achieves the fastest reward improvement and the highest final convergence value, indicating that the implicit-explicit estimation in State-IE provides a more reliable motion-state representation and improves velocity tracking over complex terrains. In contrast, removing State-IE leads to a clear reward decrease. DWL adopts implicit linear velocity estimation and shows relatively weak tracking performance. Although VPIES uses explicit linear velocity estimation, it does not jointly model explicit state estimation and implicit dynamic representation, resulting in lower final performance than TSIE.

**Figure 11:**
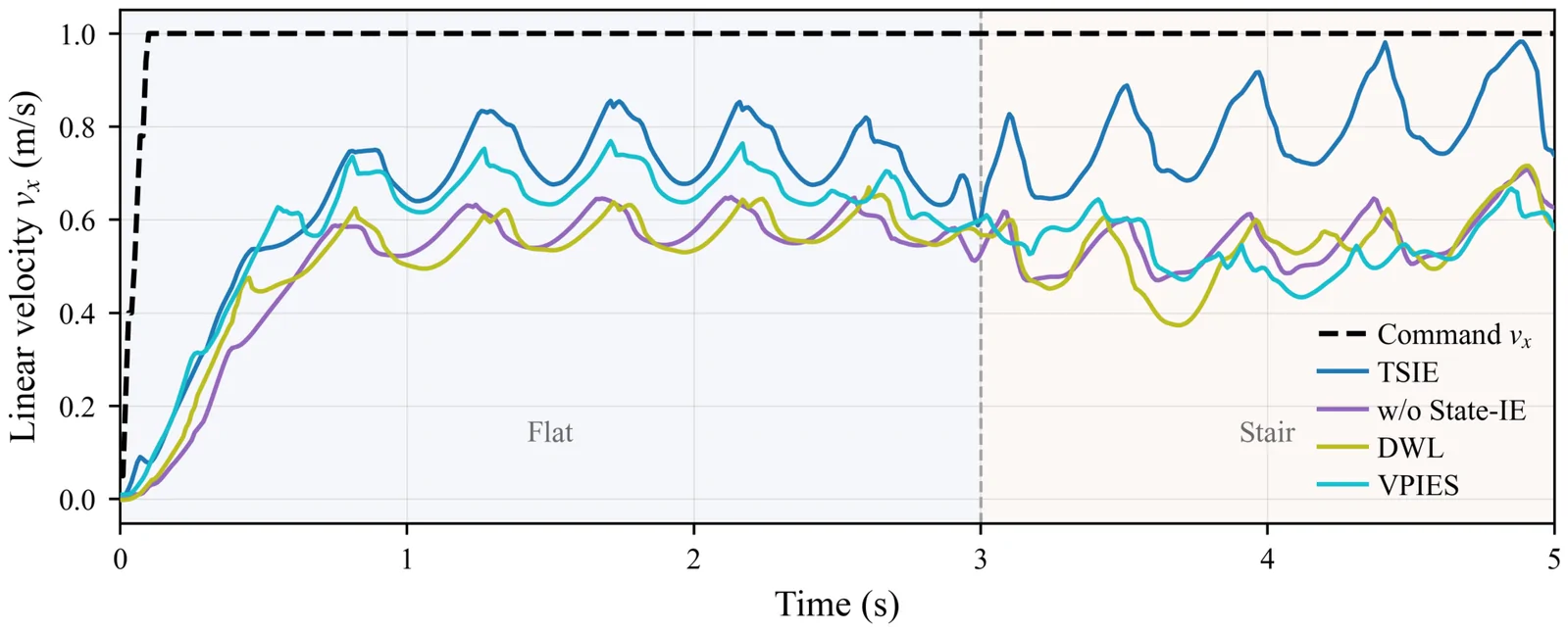
Comparison of forward linear velocity tracking curves in the flat-to-stair scenario. The interval 0–3s corresponds to flat ground, and 3–5s corresponds to 15cm stairs.

We further compare the real velocity tracking performance of different methods in the flat-to-stair transition scenario. Figure 11 shows the actual forward velocity curves under a 1.0m/s forward velocity command. The robot enters the stair terrain from flat ground at approximately 3s.The results show that TSIE achieves a faster velocity response during the flat-ground stage. After entering the stair stage, although the velocity briefly decreases when the robot first contacts the stairs, TSIE quickly recovers and maintains stable tracking. In contrast, w/o State-IE, DWL, and VPIES exhibit more pronounced velocity fluctuations and tracking deviations, indicating that State-IE enhances the policy’s adaptability to contact transitions and dynamic disturbances.

**Figure 12:**
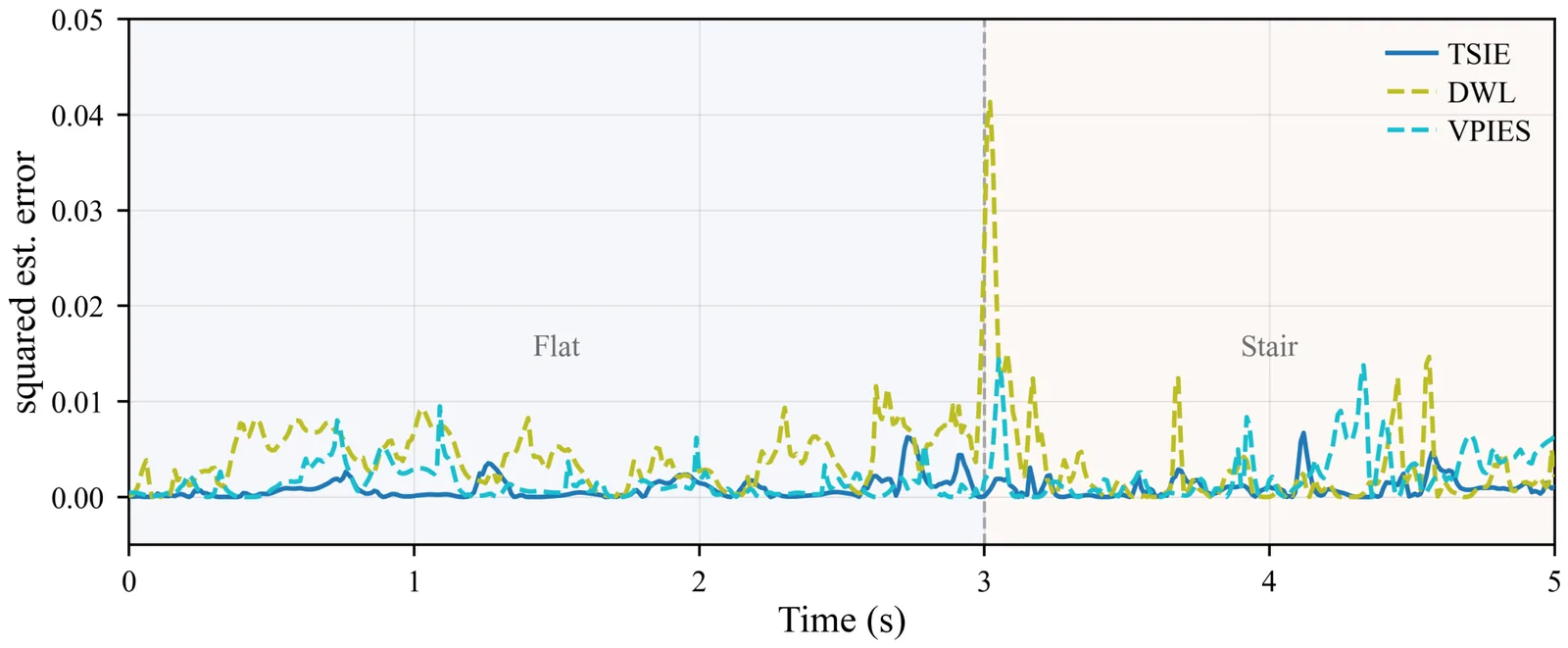
Comparison of squared forward velocity estimation errors in the flat-to-stair scenario.

Figure 12 further compares the squared forward velocity estimation errors of TSIE, DWL, and VPIES in the same scenario. Since w/o State-IE does not output velocity estimation, it is excluded from this comparison. The results show that TSIE maintains the lowest and most stable estimation error in both the flat-ground and stair stages, and effectively suppresses error fluctuations after entering the stairs. In contrast, DWL exhibits a clear error peak during the terrain transition, while VPIES, despite using explicit velocity estimation, still produces higher errors than TSIE. These results demonstrate that State-IE can provide more accurate and stable velocity-state feedback under complex contact changes by combining explicit velocity estimation with implicit dynamic representation.

**Figure 13:**
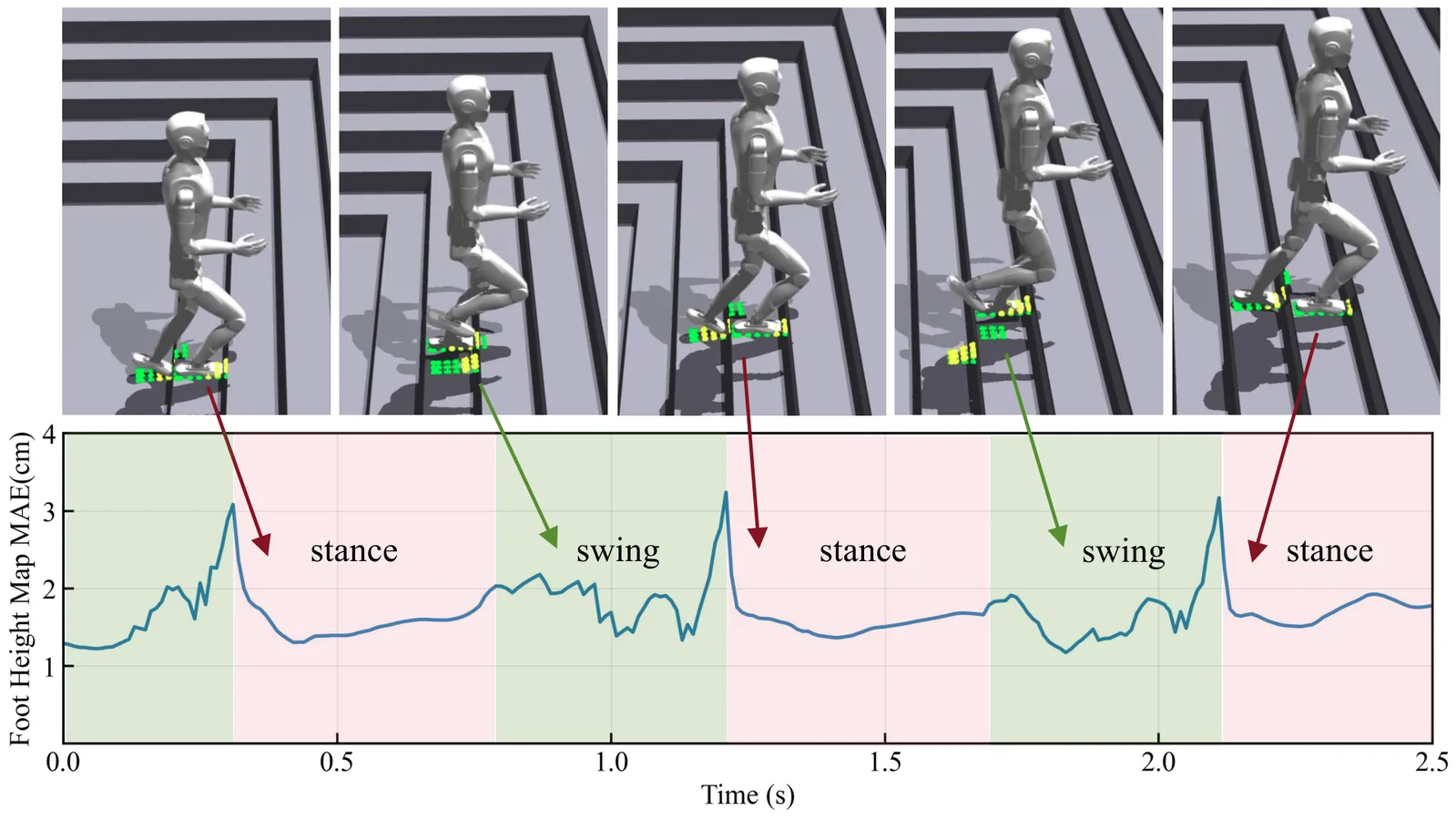
Foot height map estimation error during stair locomotion. The upper row shows representative snapshots of the robot in alternating stance and swing phases, while the lower plot reports the temporal evolution of the foot height map MAE.

To further evaluate the estimation accuracy of the foot-centered height map under high-impact conditions, Figure 13 presents the foot height map MAE during stair ascent. The upper row shows representative snapshots during the stance and swing phases, while the lower plot illustrates the corresponding MAE over time. As expected, the estimation error exhibits brief increases during the stance phase, particularly at foot-ground impact, due to abrupt contact dynamics. However, the error rapidly decreases after the contact is stabilized and remains below approximately 3.2 cm throughout the locomotion process. In contrast, the MAE stays consistently low during the swing phase. These results demonstrate that State-IE can reliably estimate the local terrain geometry around the foot using only historical proprioceptive observations, without noticeable estimation drift even under high-impact contact conditions, thereby validating the robustness and stability of the proposed method.

**Figure 14:**
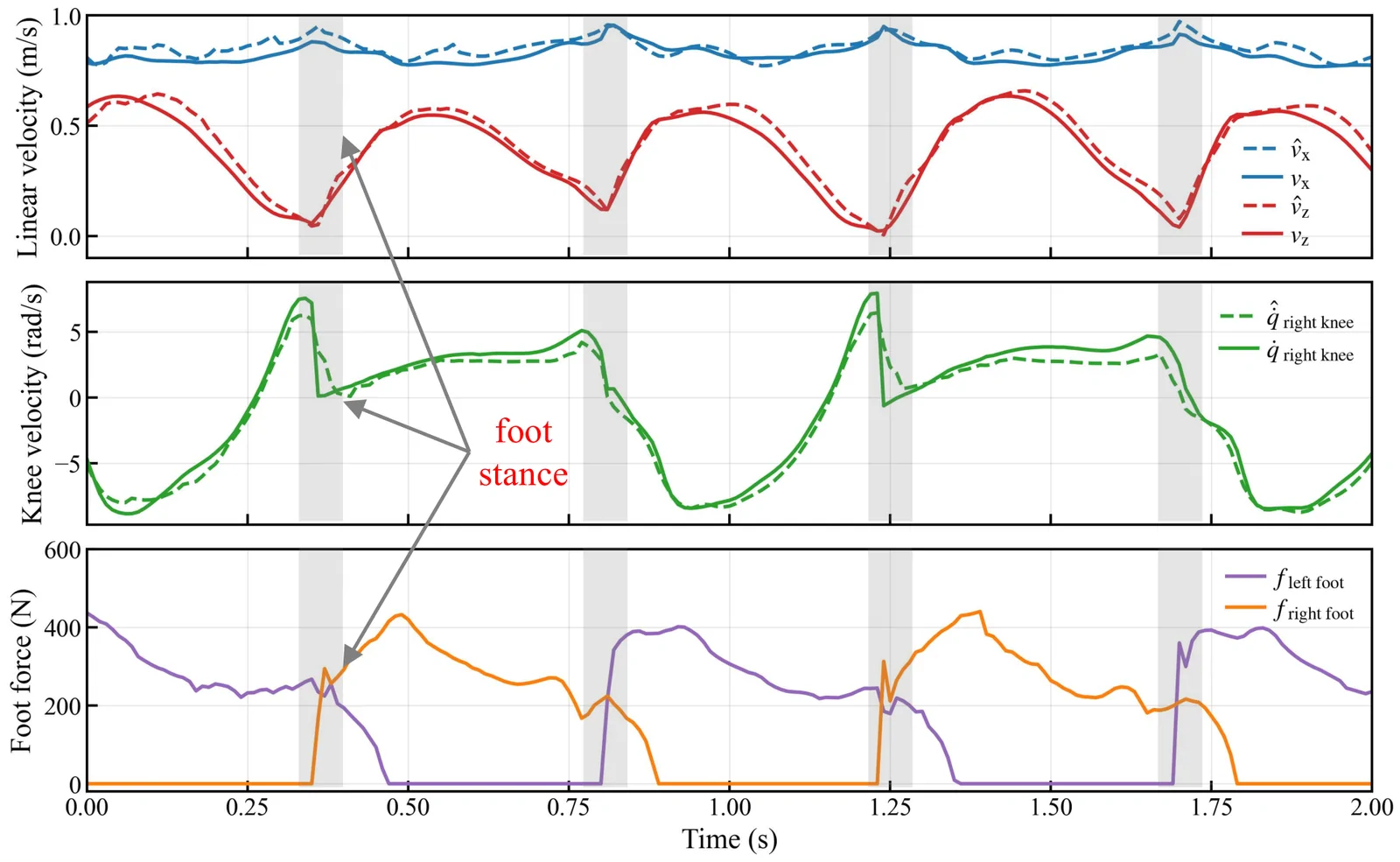
Estimated base linear velocity, predicted knee joint velocity, and foot contact force during stair ascent. The gray shaded regions indicate the foot contact transition phase. State-IE accurately captures the rapid dynamics during foot-ground contact and stance transitions, without noticeable peak attenuation or response delay.

To further evaluate the capability of State-IE in modeling the robot’s high-frequency dynamics, Figure 14 presents the estimated base linear velocity, the predicted knee joint velocity, and the foot contact forces during stair ascent. Specifically, the top panel compares the estimated and ground-truth base linear velocities, the middle panel compares the predicted and ground-truth knee joint velocities, and the bottom panel shows the contact forces of both feet. The gray shaded regions indicate the contact transition phase, where the foot switches from the swing phase to the stance phase. It can be observed that the estimated base linear velocity remains highly consistent with the ground truth throughout the entire stair-climbing process. Even during the contact transition, when the foot impacts the stair and the contact force rapidly increases, the estimated velocity accurately captures the rapid velocity variations induced by the contact impact. Meanwhile, the predicted knee joint velocity also closely follows the ground truth during the contact transition, without noticeable peak attenuation or response delay. These results demonstrate that State-IE effectively captures the high-frequency dynamic evolution around foot-ground contact, while maintaining accurate and robust state estimation and future state prediction under complex contact conditions.

**Figure 15:**
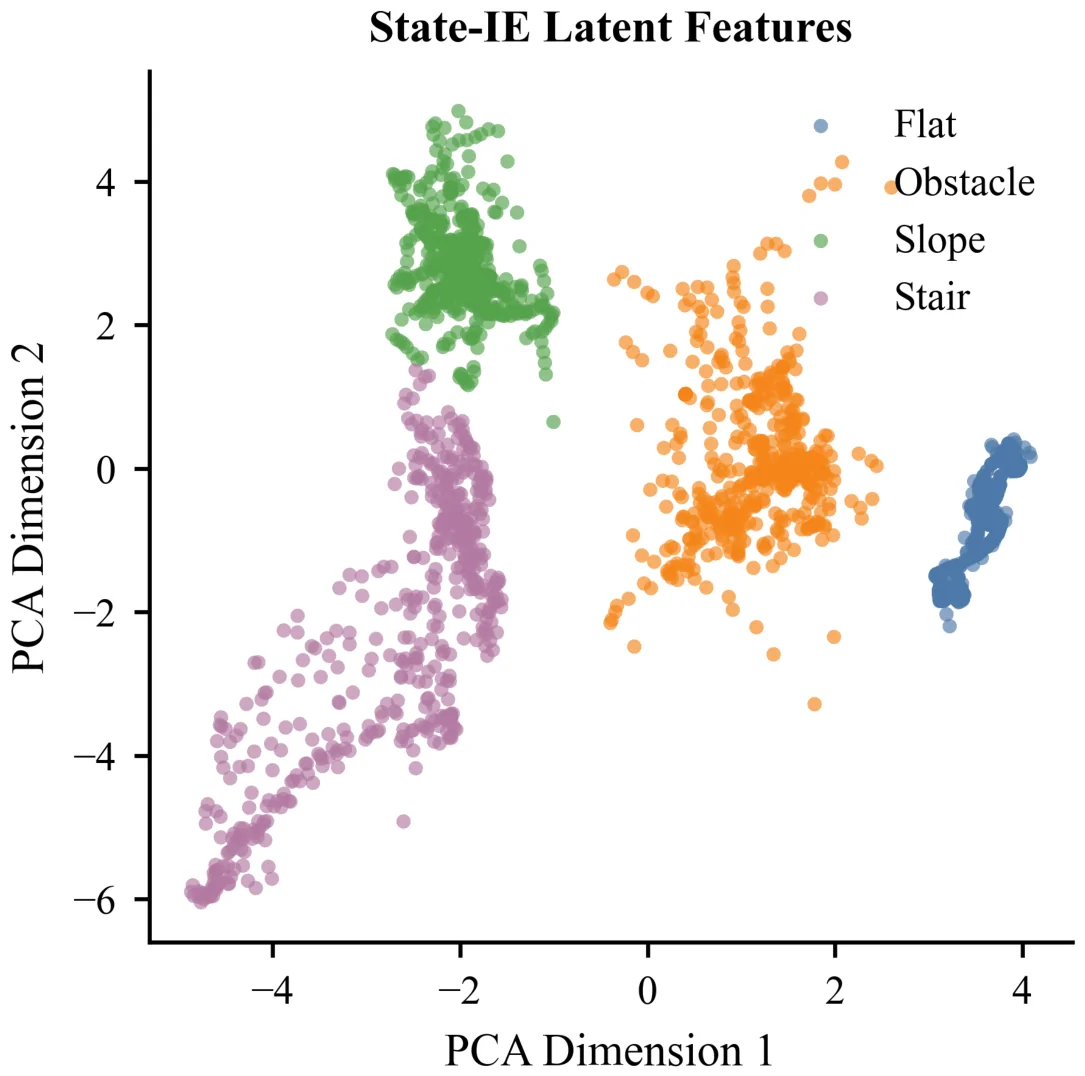
PCA visualization of the implicit latent features of State-IE on different terrains.

Finally, we visualize the latent feature vectors of State-IE using principal component analysis (PCA) to analyze its implicit terrain representation capability. Figure 15 shows the distribution of state features when the robot moves forward on different terrains for 5s. The results show that different terrain types exhibit certain clustering patterns in the latent space, indicating that State-IE can extract implicit representations related to terrain geometry, contact states, and external disturbances from historical proprioceptive information and future proprioceptive observation reconstruction.

Overall, State-IE provides the policy with more reliable state information through the synergy between explicit state estimation and implicit dynamic representation, thereby improving velocity tracking, locomotion stability, and environmental adaptability in blind locomotion over complex terrains.

### 4.3. Real-World Experiments

**Figure 16:**
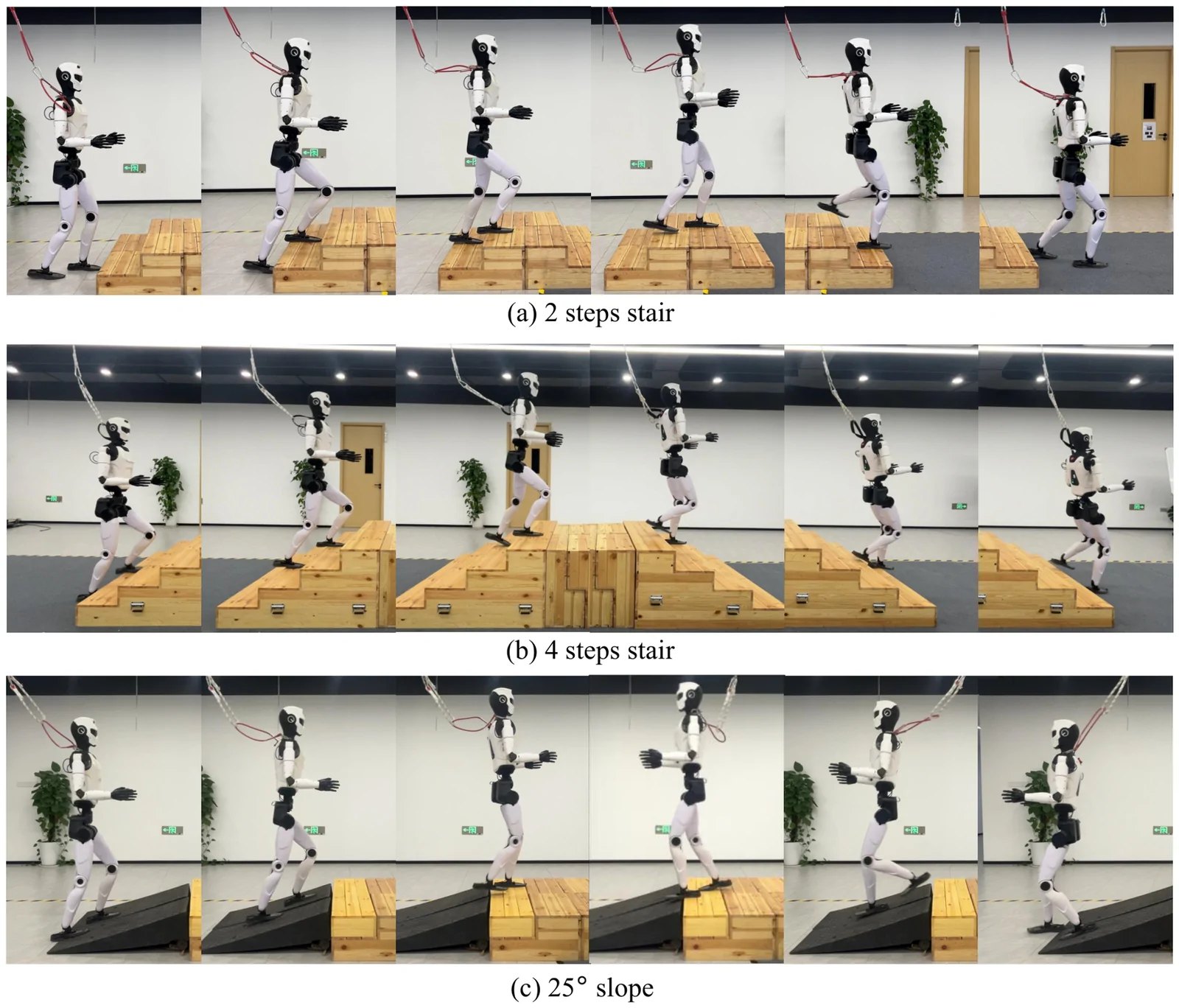
Real-world indoor terrain test scenarios for evaluating policy stability and generalization. The test scenarios include 2-step and 4-step stairs, as well as a $25^{\circ }$ slope.

We construct a real-world indoor terrain test scenario, as shown in Figure 16, to evaluate the terrain traversal capability and locomotion stability of TSIE on the physical robot platform. The test scenario includes three representative terrains: two stair configurations with different difficulty levels—a low-difficulty configuration with two consecutive steps and a high-difficulty configuration with four consecutive steps—and a $25^{\circ }$ slope. Each stair step has a height of 15cm and a width of 30cm. Compared with simulation, real-world terrain traversal involves foot-contact uncertainty, ground-friction variation, actuator latency, and modeling errors, thereby providing a more practical evaluation of the policy’s real-world adaptability and sim-to-real transfer performance.

Each method is evaluated in 20 independent trials for each terrain. A trial is considered successful if the robot traverses the corresponding terrain without falling, obvious instability, or premature termination. Table 10 reports the numbers of ascent failures, descent failures, and the success rates of different methods in the real-world terrain scenarios. The results show that TSIE achieves the highest success rate of 1.0 in both the two-step stair and the $25^{\circ }$ slope scenarios. The remaining methods also achieve relatively high success rates, and the overall trend is generally consistent with the simulation results, indicating that most policies exhibit favorable sim-to-real transfer capability on low-difficulty real stair and slope terrains. However, when the difficulty increases to four consecutive steps, the performance gap among different methods becomes more pronounced. TSIE maintains a success rate of 0.95, with only one descent failure, showing the best real-world robustness. In contrast, the success rates of w/o State-IE and VPIES decrease to 0.60 and 0.65, while that of DWL further decreases to 0.55. These results indicate that consecutive stair traversal introduces stronger contact uncertainty, accumulated foot-placement errors, and velocity disturbances, thereby imposing higher requirements on terrain representation and state estimation. Due to insufficient terrain estimation or state modeling capability, other methods are more prone to failures such as hitting stair edges, foot slippage, and unsuccessful support transitions. In contrast, TSIE provides more reliable terrain and state representations through long-horizon proprioceptive history encoding, Terrain-IE, and State-IE. This enables the robot to maintain more stable velocity tracking and foot-contact behavior during real-world stair traversal, thereby improving real-world stability and generalization in blind locomotion over complex terrains.

**Figure 17:**
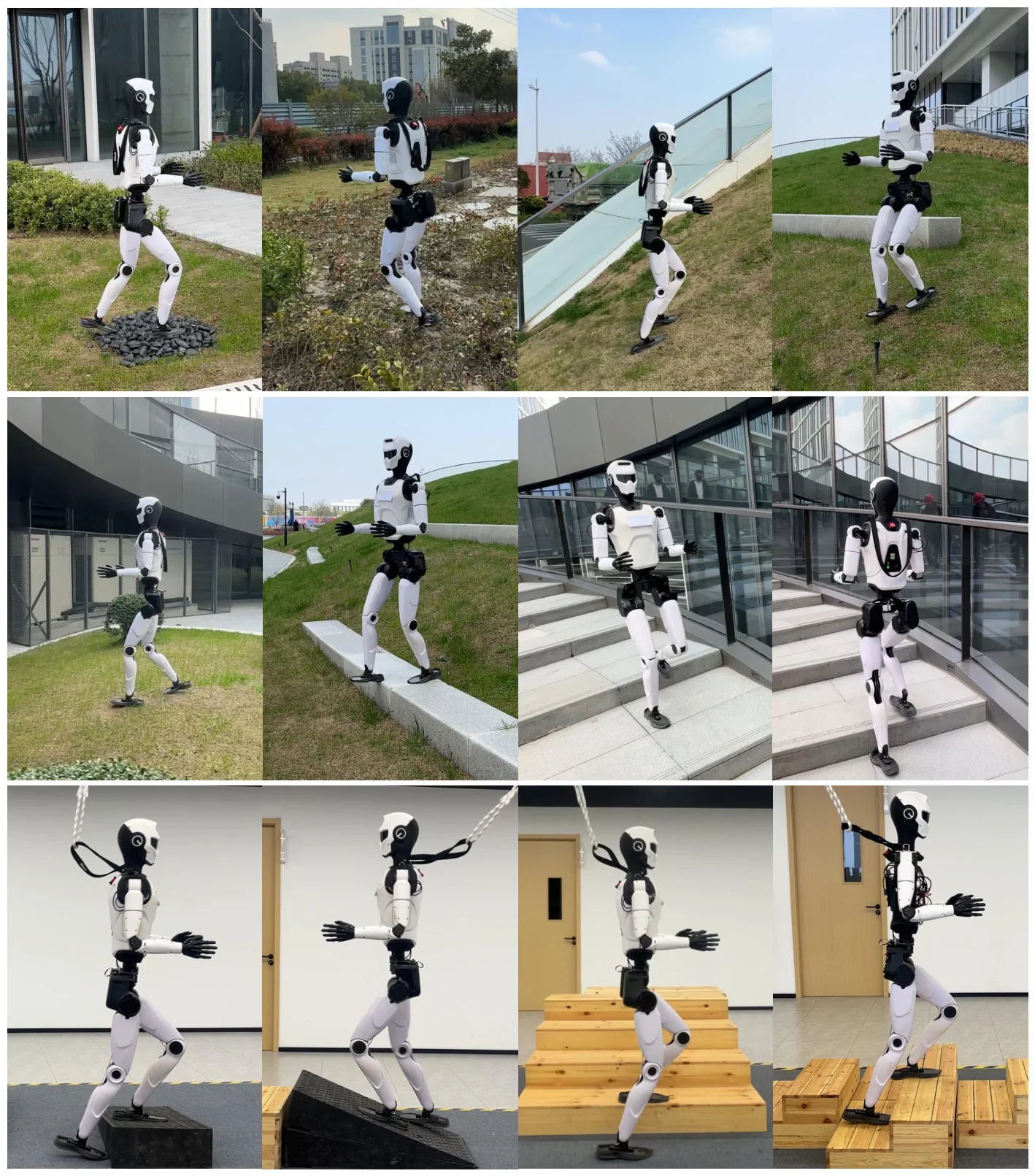
Indoor and outdoor unseen terrain scenarios used to evaluate the zero-shot generalization capability of TSIE. All experiments were conducted without additional training or fine-tuning. The test environments include slopes, stairs, grass, gravel, stone steps, and discrete obstacles.

To further validate the generalization capability of TSIE in real-world complex environments, we conduct extended walking tests in multiple indoor and outdoor scenarios, as shown in Figure 17. The test scenarios cover a variety of representative real-world terrains, including indoor $25^{\circ }$ slopes and irregular steps, as well as outdoor grass, mud, slopes, gravel surfaces, 12cm steps, and 25cm discrete obstacles.The experimental results show that TSIE enables stable locomotion across these real-world terrains and maintains favorable body posture and foot-contact stability during abrupt terrain transitions. Notably, all real-world experiments were conducted by directly deploying the same policy trained entirely in simulation, without any additional training, fine-tuning, or parameter adaptation. Real-world terrains such as grass, mud, and gravel were not explicitly modeled during training, yet the robot was still able to achieve stable locomotion using only proprioceptive information. These results further demonstrate the zero-shot generalization capability of TSIE to previously unseen real-world environments, as well as its strong sim-to-real transfer performance.

## 5. Conclusions

To address the challenges of missing environmental information, insufficient state estimation accuracy, and limited terrain adaptability in blind bipedal locomotion over complex unstructured terrains, we propose TSIE, an end-to-end reinforcement learning framework based on Terrain and State Implicit-Explicit Estimation. TSIE relies solely on historical proprioceptive information and extracts implicit dynamic features from robot–environment interactions through long-horizon history encoding. It further introduces two estimation branches, Terrain-IE and State-IE. Terrain-IE combines local explicit terrain estimation with global implicit terrain reconstruction to enhance the policy’s perception of complex terrain geometry, while State-IE integrates explicit linear velocity estimation and implicit dynamic representation to improve the modeling of robot motion states, contact transitions, and external disturbances. In addition, AdaSmpl adaptively adjusts the use of ground-truth and estimated terrain during training, improving training stability and the generalization capability of terrain representations.Simulation results show that TSIE outperforms baseline methods and ablation variants in terms of training reward, terrain curriculum level, complex-terrain traversal success rate, and stair traversal stability. Further analyses of terrain estimation, velocity tracking, and latent features demonstrate that TSIE can effectively extract implicit information related to terrain geometry, contact states, and dynamic disturbances from historical proprioceptive information, thereby improving locomotion robustness and environmental adaptability over complex terrains.Real-world robot experiments further validate the sim-to-real transfer capability of TSIE. The robot achieves stable locomotion over various real terrains, including indoor stairs, slopes, grass, gravel, mud, steps, and discrete obstacles. In particular, in challenging consecutive stair scenarios, TSIE effectively reduces failures such as hitting stair edges, foot slippage, and unsuccessful support transitions, showing strong real-world stability and robustness. Overall, by integrating long-horizon proprioceptive history encoding, multi-scale terrain implicit-explicit estimation, state implicit-explicit estimation, and adaptive training, TSIE improves environmental perception, state estimation accuracy, and locomotion robustness for blind bipedal locomotion over complex unstructured terrains.Although TSIE shows favorable stability and generalization capability, it still relies mainly on foot-contact feedback to infer local terrain structures in scenarios with abrupt height changes, such as consecutive stairs, due to the absence of exteroceptive perception. Therefore, there remains a risk of foot collisions with stair edges. Future work will consider incorporating exteroceptive modules such as depth cameras and integrating them with the proprioceptive estimation framework to enhance anticipatory perception of upcoming terrain structures, further improving foothold safety and long-term autonomous locomotion over complex unstructured terrains.

## Figures and Tables

**Table 1:** Summary of the Observation Space the Observation Space.

Component	Dim.	Observation	State
Clock input	2	✔	✔
Command	3	✔	✔
Joint position	10	✔	✔
Joint velocity	10	✔	✔
Previous action	10	✔	✔
Angular velocity	3	✔	✔
Euler angle	2	✔	✔
Base linear velocity	3		✔
Local high-res. height map	169		✔
Global low-res. height map	169		✔
Friction coefficient	1		✔
Push force	2		✔
Body mass	1		✔

✔ indicates that the corresponding component is included in the observation or state.

**Table 2:** Summary of Reward Function.

Reward Term	Equation	Weight
Lin. velocity tracking	$\exp\left(-4{∥v_{xy}^{\mathrm{cmd}}-v_{xy}∥}^{2}\right)$	1.5
Ang. velocity tracking	$\exp\left(-4{\left(\omega_{z}^{\mathrm{cmd}}-\omega_{z}\right)}^{2}\right)$	1.0
Base height(w.r.t. feet)	${\left(h_{\mathrm{target}}-h\right)}^{2}$	−20.0
Linear velocity (*z*)	$v_{z}^{2}$	−1.0
Angular velocity (xy)	$∥\omega_{xy}{∥}^{2}$	−0.5
Projected gravity	$∥g_{xy}{∥}^{2}$	−5.0
Action rate	$∥a_{t}-a_{t-1}{∥}^{2}$	−0.02
Joint torque	${∥\tau ∥}^{2}$	−2 × 10^−5^
Joint velocity	$∥\dot{q}{∥}^{2}$	−1 × 10^−3^
Joint acc.	$∥\ddot{q}{∥}^{2}$	−2.5 × 10^−7^
Feet acc.	$\sum_{f=1}^{2}∥{\dot{v}}_{f}∥$	−5 × 10^−3^
Joint velocity limit	$\sum_{i=1}^{10}\max(|{\dot{q}}_{i}|-{\dot{q}}_{\mathrm{limit}},0)$	−0.5
Joint torque limit	$\sum_{i=1}^{10}\max(|{\dot{\tau }}_{i}|-{\dot{\tau }}_{\mathrm{limit}},0)$	−0.1
Hip pos	$\sum_{i=1}^{4}{\left(q_{i}-q_{i}^{\mathrm{default}}\right)}^{2}$	−2.0
Feet air time	$\sum_{f=1}^{2}\min\left(t_{f}^{\mathrm{feet}\mathrm{in}\mathrm{air}},0.5\right)$	0.5
Feet gait contact	$r_{\mathrm{contact}}$	−2.0
Feet gait swing	$r_{\mathrm{swing}}$	−2.0
Feet slide	$\sum_{f=1}^{2}∥v_{f,xy}∥\cdot I\left(F_{f}>1\right)$	−0.2
Feet contact force	$\sum_{f=1}^{2}\max{\left(∥F_{f}∥-500,0\right)}^{2}$	−0.002
Feet stumble	$I(∥F_{f,xy}∥>2\cdot |F_{f,z}|)$	−5.0
Termination	$I_{\mathrm{terminated}}$	−200.0

**Table 3:** Module architecture with hidden layer configurations.

Module	Type	Hidden Layers	Output
Actor	MLP	[512, 256, 128]	$a_{t}$
Critic	MLP	[512, 256, 128]	$V_{t}$
History Encoder	LSTM	[128]	$z_{t}^{prop}$
Terrain-IE Encoder	MLP	[256, 128]	${\hat{h}}_{t}^{fine}$, $z_{t}^{rough}$
Terrain-IE Decoder	MLP	[64, 128]	${\hat{h}}_{t}^{rough}$
State-IE Encoder	MLP	[256, 128]	${\hat{v}}_{t}$, ${\hat{h}}_{t}^{foot}$, $z_{t}$
State-IE Decoder	MLP	[64, 128]	${\hat{o}}_{t+1}$

**Table 4:** PPO parameters configuration.

Parameter	Value
Number of environments	4096
Batch size	4096 × 24
Learning rate	adaptive
Entropy coefficient	0.01
Discount factor (γ)	0.996
GAE discount factor (λ)	0.95
Number of mini batches	4
Number of learning epochs	5

**Table 5:** Domain randomization parameters.

Terms	Range	Unit
Joint Position	[−0.05, 0.05]	rad
Joint Velocity	[−1.0, 1.0]	rad/s
Euler Angle	[−0.05, 0.05]	rad
Angular Velocity	[−0.3, 0.3]	rad/s
Payload Mass	[−4.0, 4.0]	kg
Center of Mass Offset	[−0.05, 0.05]	m
Push Velocity	[−1.5, 1.5]	m/s
Continuous Push Force	[−10.0, 10.0]	N
Ground Friction	[0.1, 1.5]	-
Ground Restitution	[0.0, 0.5]	-
Motor Strength	[0.8, 1.2]	-
Motor $K_{p}$	[0.8, 1.2]	-
Motor $K_{d}$	[0.8, 1.2]	-
System Latency	[0.0, 20.0]	ms

**Table 6:** Success rate comparison in Simulation.

Method	Success Rate							
	Stair			Slope		Discrete		
	10 cm	15 cm	20 cm	0.3 rad	0.5 rad	10 cm	15 cm	20 cm
TSIE	**0.99**	**0.97**	**0.94**	**1.00**	**0.99**	**0.99**	**0.98**	**0.92**
w/o Long History Obs	0.98	0.94	0.83	**1.00**	0.98	0.97	0.92	0.84
w/o ${\hat{h}}_{t}^{fine}$	0.98	0.93	0.90	**1.00**	**0.99**	0.98	0.90	0.89
w/o ${\hat{h}}_{t}^{rough}$	**0.99**	0.94	0.85	**1.00**	0.98	**0.99**	0.93	0.83
w/o State-IE	0.97	0.89	0.80	0.99	0.96	0.95	0.85	0.77
DWL	0.94	0.86	0.72	0.99	0.95	0.92	0.81	0.69
VPIES	0.97	0.91	0.82	**1.00**	**0.99**	0.96	0.89	0.80

Bold values indicate the best performance in each column.

**Table 7:** Stair Failures in Ascent and Descent.

Method	Stair (10 cm)		Stair (15 cm)		Stair (20 cm)	
	Up	Down	Up	Down	Up	Down
TSIE	**1**	**6**	**4**	**25**	**10**	**54**
w/o Long History Obs	3	15	13	52	28	143
w/o ${\hat{h}}_{t}^{fine}$	2	13	15	49	21	84
w/o ${\hat{h}}_{t}^{rough}$	**1**	8	12	43	30	121
w/o State-IE	8	25	27	93	47	158
DWL	17	46	34	117	65	221
VPIES	5	27	18	65	41	149

Bold values indicate the best performance in each column.

**Table 8:** Terrain Height Map Estimation Error (MAE cm).

Method	Slope Up	Slope Down	Stair Up	Stair Down
TSIE	**1.569**	**1.661**	**5.115**	**7.671**
w/o ${\hat{h}}_{t}^{rough}$	1.902	1.983	8.704	10.327
w/o AdaSmpl	1.756	1.825	6.974	8.612

Bold values indicate the best performance in each column.

**Table 9:** Sensitivity analysis of terrain height-map resolution.

Configuration	Local Resolution	Global Resolution	Terrain MAE (cm) ↓	Success Rate ↑
TSIE(5,10)	5 cm	10 cm	5.115	**0.945**
TSIE(5,15)	5 cm	15 cm	6.039	0.912
TSIE(3,10)	3 cm	10 cm	**5.107**	0.941
TSIE(10,15)	10 cm	15 cm	7.423	0.896

↓ indicates that lower values are better, whereas ↑ indicates that higher values are better. Bold values indicate the best performance for each metric.

**Table 10:** Real-world terrain traversal results of different methods, including ascent failures, descent failures, and success rates.

Method	2-Steps Stair			4-Steps Stair			$25^{\circ }$ Slope
	Up Falls	Down Falls	SR	Up Falls	Down Falls	SR	SR
TSIE	**0**	**0**	**1.00**	**0**	**1**	**0.95**	**1.00**
w/o Long History Obs	1	1	0.90	2	3	0.75	0.95
w/o ${\hat{h}}_{t}^{fine}$	1	1	0.90	2	4	0.70	0.90
w/o ${\hat{h}}_{t}^{rough}$	1	0	0.95	1	2	0.85	0.95
w/o State-IE	1	2	0.85	3	5	0.60	0.85
DWL	3	3	0.70	3	6	0.55	0.80
VPIES	2	1	0.85	2	5	0.65	0.90

Bold values indicate the best performance in each column.

## Data Availability

The original contributions presented in this study are included in the article material. Further inquiries can be directed to the corresponding author.
